# Statistical analysis supports the size control mechanism of *Chlamydia* development

**DOI:** 10.1371/journal.pcbi.1013227

**Published:** 2025-07-14

**Authors:** Jinsu Kim, Christine Sütterlin, Ming Tan, German Enciso

**Affiliations:** 1 Department of Mathematics, Pohang University of Science Technology, Pohang, Republic of Korea; 2 Department of Developmental and Cell Biology, University of California, Irvine, California, United States of America; 3 Departments of Microbiology and Molecular Genetics, and Medicine, University of California, Irvine, California, United States of America; 4 Department of Mathematics, University of California, Irvine, California, United States of America; Leiden University Faculty of Science: Universiteit Leiden Faculteit der Wiskunde en Natuurwetenschappen, NETHERLANDS, KINGDOM OF THE

## Abstract

*Chlamydia* is an intracellular bacterium that reproduces via an unusual developmental cycle that only occurs within a eukaryotic host cell. A replicating form of the bacterium (RB) repeatedly divides to produce about a thousand progeny, which convert in a delayed and asynchronous manner into the infectious form (EB). The regulatory mechanisms that control this developmental switch are unknown, but they could potentially include extrinsic signals from the host cell or other chlamydiae, or an intrinsic signal such as chlamydial cell size. In this paper, we investigated the regulation of RB-to-EB conversion by developing and analyzing three mathematical models, each based on a different regulatory mechanism. To test these models, we derived statistical evidence from parameters, including number, size and location of RBs and EBs, obtained from experimental measurements and model fitting. All three models successfully reproduced the experimentally measured timing of RB-to-EB conversion and growth curves of the developmental forms in an infected cell. However, only the size control model, which postulates that RB size is an intrinsic signal that regulates the timing of RB-to-EB conversion, reproduced two additional statistical properties of the intracellular infection. These properties are a positive correlation between the number of RBs and EBs throughout the developmental cycle and the monotonic evolution of the coefficient of variation of EB number. This analysis thus provides support for the size control model.

## Author summary

*Chlamydia trachomatis* is a bacterium that infects mammalian cells and reproduces inside them before invading other cells. Before it can leave its mammalian host cell, it must first convert into a spore-like form called elementary body (EB), which is able to survive outside the host but cannot reproduce. Conversion into this form is a crucial cell fate decision for *Chlamydia*, and in this work we aim to compare different possible mechanisms that might regulate and drive this decision. Specifically, we use a statistical analysis of the timing of conversion, to rule out a broad series of possible mechanisms. We argue that any mechanism that has an external input for conversion will tend to feature a negative correlation between unconverted and converted *Chlamydia* forms. However, the experimental data indicates a positive correlation between the forms. We conclude that a mechanism internal to each chlamydia is more likely at work. This supports a so-called size control mechanism, where *Chlamydia* converts once it reaches a sufficiently small size after multiple divisions. An additional analysis of the noise in the number of EB forms for each inclusion further supports this conclusion.

## Introduction

*Chlamydia* is a genus of pathogenic bacteria that cause significant infections in humans and animals. The most clinically important *Chlamydia* spp. is *C. trachomatis*, which is the most common cause of bacterial sexually transmitted infection [1]. *C. trachomatis* also causes an ocular infection that can lead to blindness, and a related species *C. pneumoniae* is a common cause of community-acquired pneumonia. More cases of *Chlamydia* infection are reported to the CDC each year than all other infectious diseases combined (CDC, 2019).

All *Chlamydia* spp. are obligate intracellular bacteria that only reproduce within a eukaryotic host cell. The hallmark of the intracellular *Chlamydia* infection is its developmental cycle that occurs over 48–72 hours and involves conversion between two specialized developmental forms of the bacterium. The elementary body (EB) is the infectious form, which enters an epithelial host cell and converts into the metabolically active reticulate body (RB) inside a membrane-bound compartment called the chlamydial inclusion. The RB repeatedly divides but starting at about 24 hours post infection (hpi), a developmental switch takes place in which individual RBs convert into an EB. RB-to-EB conversion is critical because only EBs can spread the infection to a new host cell, but the regulatory signals that control this differentiation step are not known.

Mathematical modeling has been used to study specific aspects of human *Chlamydia* infection, including transmission [2], immunity [3], vaccination [4] and screening [5]. There has been more limited use of this modeling approach to investigate the intracellular infection. The few published studies have mainly focused on RB-to-EB conversion because it is a critical control step in the developmental cycle. Wilson, Bavoli and colleagues proposed a *contact-dependent model* in which RB-to-EB conversion is negatively controlled by contact between each RB and the inclusion membrane [6,7]. Early in the intracellular infection, the inclusion is small and contact between the RB and the inclusion membrane inhibits conversion. At later times, when the inclusion has grown, RBs are hypothesized to lose contact with the inclusion membrane, which allows conversion into an EB to occur. This model is supported by mathematical modeling and electron micrograph observations showing that RBs are predominantly located at the periphery of the chlamydial inclusion [8]. An alternative hypothesis was proposed by Omsland and colleagues, based on the idea that nutrient availability and depletion ultimately drive conversion to EB [9,10].

The *size control* model, developed by our own group, proposes that an RB can only convert into an EB when it is smaller than a threshold size [11]. This model is supported by three-dimensional electron microscopy (3D-EM) analysis of *C. trachomatis*-infected cells, which measured a progressive 6-fold reduction in average RB volume over the course of the developmental cycle and only detected RB-to-EB conversion when average RB size was small. Thus, RB size may function as an intrinsic, cell autonomous signal for RB-to-EB conversion, whereas the contact-dependent and nutrient models rely on extrinsic signals, outside the individual bacterium, to regulate this important step in the chlamydial developmental cycle.

Chiarelli et al. used mathematical modeling and experimental measurements to propose that RB-to-EB conversion is controlled by an intrinsic, rather than extrinsic, signal [12]. They used two different deterministic models, one with an external environmental signal common to all chlamydiae and one with a signal inside each chlamydia, and they tested different parameters of the models to differentiate the behavior of the two models. They then carried out fluorescence and live-cell imaging experiments to test the model predictions and concluded that the signal is likely intrinsic and is affected by RB growth and division rates. Notice that since their models are deterministic, they are unable to use stochastic information such as correlation between different measurements that is directly available from experiments. Some of their model assumptions, such as the consumption of the signal by the RB, are compatible with the nutrient availability mechanism but do not seem as readily applicable to mechanisms such as the contact-dependent model.

This same group recently published a *Chlamydia* modeling paper focused on the potential role of asymmetric division [13]. The authors included stochastic effects by using two agent-based models with or without asymmetric division elements. They conclude that the mechanism most consistent with experimental data is an asymmetric model in which there is an initial period of symmetric replication, followed by asymmetric RB division giving rise to one RB and one IB per division cycle. In our own experimental work, we have not observed chlamydial developmental forms undergoing asymmetric division. Furthermore, this model predicts that the RB population size will be maintained over time, but we measured a decrease in the number of RBs late in the infection [11]. For this reason, we do not focus our attention on this particular question and assume for simplicity that division is symmetric.

Finally, Wan, Enciso and colleagues [14–16] proposed linear and non-linear growth models to study the optimal proliferation and differentiation timing of *Chlamydia*. Through an optimal control argument, they showed that the switch from RB replication to RB-to-EB conversion should be a form of *bang-bang control* in order to maximize EB production. Bang-bang control refers to a behavior that optimizes a given output by switching suddenly from one extreme mode to another, in this case from replication to conversion.

In the current paper, we use a chemical reaction network framework to compare different mechanisms for the regulation of the chlamydial developmental cycle. We consider three potential models for the regulation of RB-to-EB conversion. The first model is a recapitulation of the contact-dependent hypothesis as proposed by Wilson and colleagues and described above. A second model, referred to as the *communication model, is analogous to nutrient models and* is loosely based on the bacterial quorum sensing mechanism in which bacteria in a population produce and respond to a signaling molecule as a form of intra-bacterial communication [17]. Notably, the signal we use in this model is not consumed, in contrast to the nutrient model. The third model is a simplified form of the size control mechanism, as proposed in [11].

We used statistical quantities to qualitatively validate and compare these three models with experimentally measured data from Lee et al. To facilitate this comparative analysis, we used a unified framework for the three models based on chemical reaction networks and stochastic modeling. We found that the RB/EB correlation based on experimental measurements was positive throughout the developmental cycle. This correlation coefficient was initially positive for all three models but turned negative at 40 hours post infection (hpi) for both the communication models and contact-dependent, while it remained positive for the size control model. This change in the correlation coefficient is a consistent qualitative feature of the communication and contact-dependent models that is conserved under most parameter values. We also observed that the coefficient of variation of EB number decreased in a broadly monotonic manner for the experimental datasets and the size control model, but that it showed non-monotonic behavior for the contact dependent and communication models.

This manuscript is outlined as follows. We describe the three mathematical models in detail, including the background assumptions in each case. We then show how these three stochastic models have different statistical features when compared to the experimental measurements. In particular, we used the RB/EB correlation and the coefficient of variation of the EB numbers to compare and contrast the three models. For each statistical comparison, a theoretical validation is also implemented using mathematical analysis.

## Experimental dataset

The main dataset produced in the work by Lee et al. [11] was derived from 3-dimensional EM images of the chlamydial inclusion and its content of chlamydial forms in *C. trachomatis*-infected human cells. Nine inclusions were analyzed for each timepoint, and for each inclusion, four different developmental chlamydial forms (RBs, EBs, as well as two intermediate forms: dividing RB (DB) and an intermediate body (IB), which is an RB in the process of converting into an EB) were distinguished and quantified. In addition, the average size of each developmental form was measured for each inclusion. Inclusions were analyzed at 4-hour time points from 12 hours post infection (hpi) to 40 hpi, which covers the periods of RB replication and RB-to-EB conversion during the intracellular infection.

The total number of chlamydiae measured in an inclusion increased from one or two at 12 hpi to around 100 at 24 hpi and around 1,000 at 40 hpi. Due to the large number of chlamydiae, a limited number of inclusions were imaged and analyzed at each time point. The volume of each inclusion was also measured, and there was a strong linear correlation between inclusion volume and the total number of chlamydiae per inclusion throughout the intracellular infection. The original study did not perform a correlation analysis of the different developmental forms, but we used the raw data to calculate the mean and variance of the population of each chlamydial form at every time point. We provide a data table in S1 Table.

## Three mechanistic models for the regulation of RB-to-EB conversion

**Fig 1:**
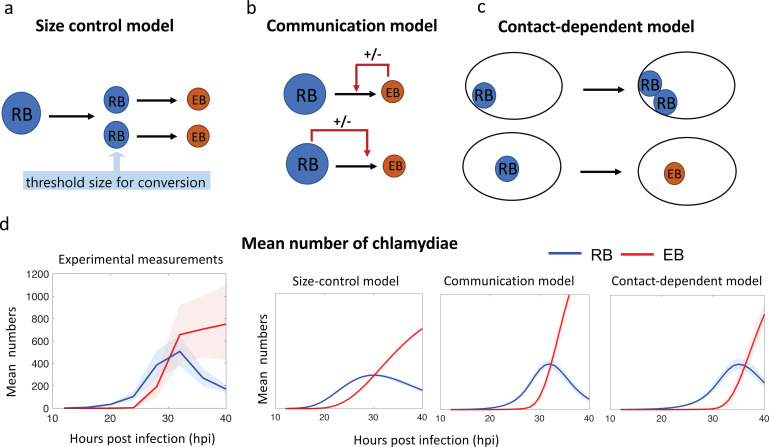
a–c. Schematic Figs showing models for three putative regulatory mechanisms controlling RB-to-EB conversion. d. Growth curves showing the mean number of RBs (blue) and EBs (red) per inclusion measured experimentally in [11] or generated by each model. For each model, the error bands (shaded area) are obtained from 100 data sets, with each data set computed from 100 independent time trajectories of the Markov models. For experimental measurements, the bootstrap algorithm is used to reproduce 100 data sets for each time point. The solid lines are the mean of the 100 mean time trajectories obtained with the data sets.

We developed a stochastic mathematical model for each of the three proposed mechanisms (Fig 1). To limit the modeling and analysis to the two main developmental forms, RBs and EBs, we included DBs and IBs with the RB and EB populations, respectively.

We used the formalism of chemical reaction network theory [18] for each of the models, where RBs and EBs are treated as species, and RB division, RB growth and RB-to-EB conversion are modeled as reactions. Since the species in question are in very low quantity, we used a stochastic formalism to describe the resulting dynamics of individual copies of each species, using continuous-time Markov chains [19], and we simulated them using the Gillespie algorithm [20,21]. The model parameters are fitted by the simulated annealing algorithm, where we searched parameters with random perturbation and accepted the new searched parameters with an acceptance probability defined by the change of the loss function [22–24]. See Section G of S1 Text for details of this algorithm. In addition, as will be shown below, we will not limit our analysis to the fitted parameters, but we will also test each of the three models with more than $10^{5}$ different parameter sets (see Section G of S1 Text). Additional details about reaction networks, their associated dynamical systems, and the parameter fitting procedures are presented in the supplementary S1 Text. See Table 1 for a summary description of the three models described here.

**Table 1:** Main features of the three models.

Model Features	Size control model	Communication model	Contact-dependent model
Mechanism controlling RB-to-EB conversion	RB size controls conversion. Only a small RB can convert into an EB	A signal from an EB/RB promotes/inhibits conversion of an RB into an EB	Contact between an RB and the inclusion membrane inhibits conversion. Only an RB that has lost contact can convert into an EB
Signal type	Intrinsic	Extrinsic	Extrinsic
Reactions for the model	RB division, RB growth, and RB-to-EB conversion	RB division and RB-to-EB conversion	RB division, diffusion, and RB-to-EB conversion

For the uncertainty analysis on the intrinsic noise of the mean trajectories, we obtained error bands with the 90th percentile upper bound and the 10th percentile lower bound (the shaded areas around the solid curves) for each time evolution of the mean growth curves and the statistical features. To show uncertainty stemming from intrinsic noise of the stochastic models, we used error bands with 100 data sets, each of which is computed with 100 independent time trajectories of the Markov models (Fig 1d). The upper and lower bounds of the error bands were obtained with the 90th percentile and the 10th percentile of the 100 data sets, respectively. For the uncertainty analysis on experimental measurements in Fig 1d, we used bootstrap algorithms to generate 100 data sets for each time point (Fig 1d, left). The error bands in Fig 1d also give 90th percentile upper bound and the 10th percentile lower bound.

### Size control model

**Fig 2:**
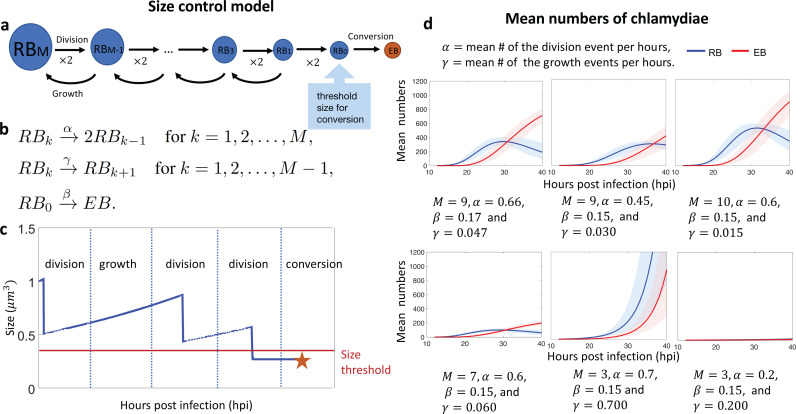
a. A schematic description of the size control model. b. A reaction network associated with the size control model. c. Cartoon illustration of the time evolution for the size of an individual RB. During the time interval indicated by vertical dotted lines, each RB can go through either i) division or ii) growth without division. Once the size of the RB reaches the size threshold, it can convert into an EB. The asterisk indicates the time point of conversion. d. Growth curves for the mean number of RBs and EBs per inclusion generated by the size control model under different parameters. We used β=0.15 for all six plots. The error bands (shaded areas) are obtained by randomly perturbing the given parameters. The solid lines are the mean of the 100 mean time trajectories obtained with the data sets.

This model proposes that 1) RB size is an intrinsic signal that regulates RB-to-EB conversion, 2) an RB can only convert into an EB below a conversion size threshold (Fig 1a and [11], and 3) RBs get smaller through division, leading to two daughter RBs whose birth size is smaller on average than the birth size of their mother. The size control model can be mathematically described with three main reactions: RB division, RB growth, and RB-to-EB conversion (Fig 2a, and reaction network in Fig 2b). We classified RBs by size into M groups: ${RB}_{k}$ for k=0,1,2,…,M, where ${RB}_{M}$ is the group with the largest RBs. For RB division, the reaction ${RB}_{k}\to {2RB}_{k-1}$, produces two daughter RBs that belong to a smaller RB group than their mother. The RB growth reaction, ${RB}_{k}\to {RB}_{k+1}$, allows an RB to shift to a larger RB group prior to division. To model the observed decrease in RB size, we allowed RB growth events to occur infrequently relative to RB division. See Fig 2c for a sample RB size time course. After repeated rounds of RB division and growth, an RB can become small enough to reach the conversion threshold size, which we defined as ${RB}_{0}$, the group with the smallest RBs. RB-to-EB conversion was modeled with the reaction ${RB}_{0}\to EB$, where EB is the number of EBs.

Fig 2d shows the mean number of RB over time ($\sum_{i=0}^{M}{RB}_{i}$). Notably, this model inherently produces a delay in the onset of RB-to-EB conversion because of the time required for RBs to undergo sufficient rounds of division to reduce their size below the conversion size threshold. In other words, the bang-bang switch from replication to conversion is modeled as the random time for ${RB}_{0}$ to be created, so that the reaction ${RB}_{0}\to EB$ can take place.

We used parameters for these reactions that were informed by experimental measurements from *C. trachomatis*-infected cells [11]. For instance, we modeled an average of ten RB divisions before conversion to produce 1,000 (2^10^) progenies, which is the mean number of chlamydiae per inclusion by the end of the intracellular infection [11]. To model the observed heterogeneity in RB size [11], we allowed each chlamydia to undergo an average of one discrete RB growth event (${RB}_{k}\to {RB}_{k+1}$) before conversion. The general shape of the experimentally measured growth curves for RBs and EBs (Fig 1d, left) is qualitatively well reproduced by multiple parameter choices with M≥7 (Fig 2d). However, the bang-bang behavior is not reproduced with small M (Fig 2d). The parameters M=9, α=0.66,β=0.17 and γ=0.047 were searched by the simulated annealing algorithm for a fit to the experimental growth curves (compare Fig 2d, top left to Fig 1d, left), and are used for the rest of the analysis of the size control model.

To show the uncertainty given by parameter perturbations, the error bands in Fig 2d were obtained by randomly varying the parameters by 20%. Notice this is a different use for error bands from that in Fig 1. For a given parameter x, we set the perturbed parameters as $x_{new}=x\left(1+U_{x}\right)$, where $U_{x}$ is an independent uniform random variable on [−0.2,0.2]. We will also follow the same parameter randomization procedure for the other models below.

### Communication model

**Fig 3:**
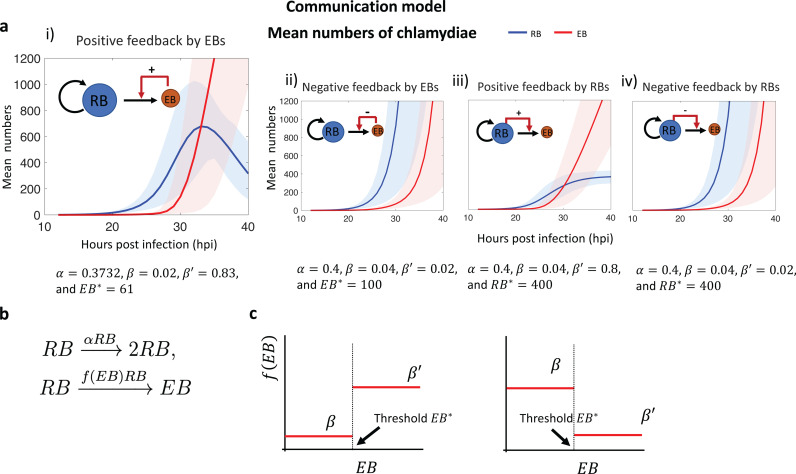
a. Growth curves showing the mean number of RBs and EBs per inclusion for each version of the communication model. The largest panel (the model with positive feedback by EBs) fit best with the growth curve of the experimental data (Fig 1d). The error bands (shaded areas) are obtained by randomly perturbing the given parameters. The solid lines are the mean of the 100 mean time trajectories obtained with the data sets. b. A reaction network description of the communication model with the extrinsic signal driven by the EB. c. Graphs of the signal functions f(EB) for EB-derived positive feedback (left) and EB-derived negative feedback (right), respectively, including the definition of the threshold value $EB^{*}$.

This model proposes that conversion of an RB into an EB is controlled by a stimulatory or inhibitory signal from another RB or an EB. For simplicity, we assume that there is only a single signal. We considered four scenarios that varied in the source (RB or EB) and effect (positive or negative) of the putative signal (Fig 3a). We quickly eliminated the two scenarios in which the RB is the source of the signal (Fig 3a iii-iv) because they produced monotonically increasing population averages in our simulations, unlike the non-monotonic RB population average found in experimental measurements (Fig 1d). Furthermore, they behaved as autonomous one-variable systems that are incapable of non-monotonic behavior because they were entirely determined by the RB itself, without input from an EB. See Section C in S1 Text for additional information on these scenarios.

We then consider the two scenarios (Fig 3a iii) in which the EB is the source of a signal that either promotes or inhibits RB-to-EB conversion. We modeled control of RB-to-EB conversion by an EB-derived signal using the reaction network scheme described in Fig 3b, where the regulatory function f(EB) depends on the number of EBs. The shape of the regulatory function was chosen for simplicity as shown in Fig 3c, using a threshold input value EB*.

We tested these two scenarios by setting the function f(EB) as either an increasing or a decreasing function. If f(EB) was a decreasing function (Fig 3c, right), there was negative feedback of RB-to-EB conversion, with inhibition of conversion as the number of EBs increases. In addition, the RB number was no longer reduced by conversion into EBs, and thus it continued to expand at later time points. This resulted in monotonically increasing time courses of RB and EB numbers (Fig 3a, ii) that did not resemble the experimentally measured growth curves. Specifically, there was no longer bang-bang control, which is characterized by a shift from RB replication to RB-to-EB conversion. We provide a more precise analysis for the negative feedback case in Section C of S1 Text.

In contrast, if f(EB) was defined as an increasing function, we observed positive feedback of RB-to-EB conversion, with an exponential increase in EB number and depletion of RBs through conversion (Fig 3a, i). This version of the communication model, with EBs providing a positive, extrinsic signal for RB-to-EB conversion, produced growth curves for the number of RB and EB that are consistent with the experimentally measured growth curves (Fig 1d). The parameters of Fig 3ai) are fitted with the stimulated annealing algorithms, and they will be used for the rest of this paper. In S1 Text we considered other forms of the signal function f(EB). In particular we show that a gradually increasing function f(EB) was able to reproduce both the bang-bang control in the mean growth curve and the non-monotonic behavior of the average RB population.

### Contact-dependent model

**Fig 4:**
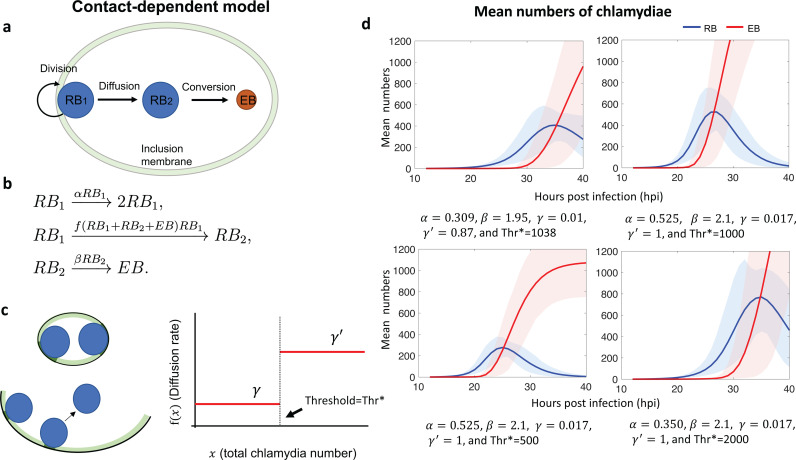
a. Schematic diagram of the contact-dependent model. b. Reaction network descriptions of the contact-dependent mathematical model. c. Left: RBs can detach from the inclusion membrane depending on the size of the inclusion membrane. Right: The graph of the signal function f(x), where x is the total chlamydial number and γ,γ′ are increasing diffusion values away from the membrane. d. Growth curves of the mean numbers of RBs and EBs with different choices of the parameters. The error bands (shaded areas) are obtained by randomly perturbing the given parameters. The solid lines are the mean of the 100 mean time trajectories obtained with the data sets.

The contact-dependent model posits that contact between an RB and the inclusion membrane inhibits RB-to-EB conversion [6,8]. The conversion rate is negligible when an RB is adjacent to the inclusion membrane, and the rate is positive if the RB is detached and in the interior of the inclusion (Fig 4a). At early times, the inclusion is not much larger than the chlamydiae within it, and the RB has extensive contact with the inclusion membrane, which limits its diffusion. However, at later times, when the inclusion volume is comparatively large, the membrane is relatively flat compared to an RB, and there is reduced physical contact between the RB and the inclusion membrane, which is presumed to allow the RB to diffuse away (Fig 4c, left).

It is important to include spatial information in any model of this hypothesis, which could be done using stochastic partial differential equations. In this paper, we use the following reaction network scheme for simplicity (Fig 4b). Let ${RB}_{1}$ indicate the population size of chlamydiae in contact with the inclusion membrane, and ${RB}_{2}$ the size of the population in the interior of the inclusion. In our reaction scheme we assumed that only ${RB}_{2}$ can convert into an EB, while only ${RB}_{1}$ can replicate. We estimated the size of each inclusion from the number of chlamydiae within the inclusion, based on the findings of Lee et al. (Lee et al., 2018), which showed a linear correlation between inclusion size and the total number of chlamydiae. In this way, as the number of chlamydiae grows, the inclusion becomes larger and increasingly allows diffusion from the membrane to the interior. We include these considerations in the model with a diffusion rate that is determined by a regulatory function $f(RB_{1}+RB_{2}+EB)$. Moreover, we define a threshold volume for diffusion, so that the diffusion rate f=γ is small for small inclusion and population sizes, and the diffusion rate f=γ ‘ is larger once a critical inclusion size is reached.

A critical part of this model is the reaction ${RB}_{1}\to {RB}_{2}$, which models diffusion of RBs from the inclusion membrane to the interior of the inclusion. Note again that diffusion is determined by the size of the inclusion, which is proportional to the total chlamydial number. Thus we set the rate of the reaction ${RB}_{1}\to {RB}_{2}$ as a step function of the chlamydial population size, which is described in Fig 4c (right). As in the communication model, we also highlight that the rate of diffusion reaction is modeled as a switch-like function. It is also controlled by the total chlamydia number, which is a chlamydia-extrinsic signal. We did not include the reverse reaction ${RB}_{2}\to {RB}_{1}$ in our model because the inclusion only gets larger over time (Lee et al., 2018), and the reduction in contact area makes it unlikely that the RB will reattach. The mean chlamydial numbers with different parameters are shown in Fig 4d. The top left graph of Fig 4d best resembled the experimental growth curves (Fig 1d), and so we used its parameter set for further analysis.

## Results

To analyze and compare the three models for the regulation of RB-to-EB conversion, we used the time course measurements of the number of RBs and EBs in an inclusion from the Lee et al. data as our experimental gold standard. For each model, we identified parameters that reproduced the experimentally measured growth curves of RB and EB number (Fig 1d). We then compared our model simulations and the experimental data using two statistical properties: 1) the correlation between the numbers of RBs and EBs within an inclusion at later times in the intracellular infection, and 2) the time evolution of the coefficient of variation of the number of EBs per inclusion.

### Positive correlation between RB and EB at all timepoints

By analyzing the 3D EM measurements from *C. trachomatis*-infected cells [11], we determined that a positive correlation coefficient between the number of RBs and EBs is maintained during the intracellular infection. Using the experimentally measured numbers of RB and EB at 4-hour intervals from 12 to 40 hpi, we empirically calculated the correlation coefficient (also called the Pearson correlation coefficient) across replicate experiments as


$$\begin{array}{c} {\hat{\rho }}_{RB,EB}(t)=\frac{\sum_{i}\left(RB_{i}(t)-\overset{―}{RB}(t)\right)\left(EB_{i}(t)-\overset{―}{EB}(t)\right)}{{\left(\sum_{i}{\left(RB_{i}(t)-\overset{―}{RB}(t)\right)}^{2}\sum_{i}{\left(EB_{i}(t)-\overset{―}{EB}(t)\right)}^{2}\right)}^{0.5}}, \end{array}$$ (1)

where $RB_{i}(t)$ and $EB_{i}(t)$ are the number of RBs and EBs at time t, respectively, in the i-th inclusion dataset. $\overset{―}{RB}(t)$ and $\overset{―}{EB}(t)$ are the sample mean of RBs and EBs at time t, respectively. For the size control model and the contact-dependent model, RB(t) is the sum of all the subclasses $RB_{i}(t{)}^{\prime }s$.

**Fig 5:**
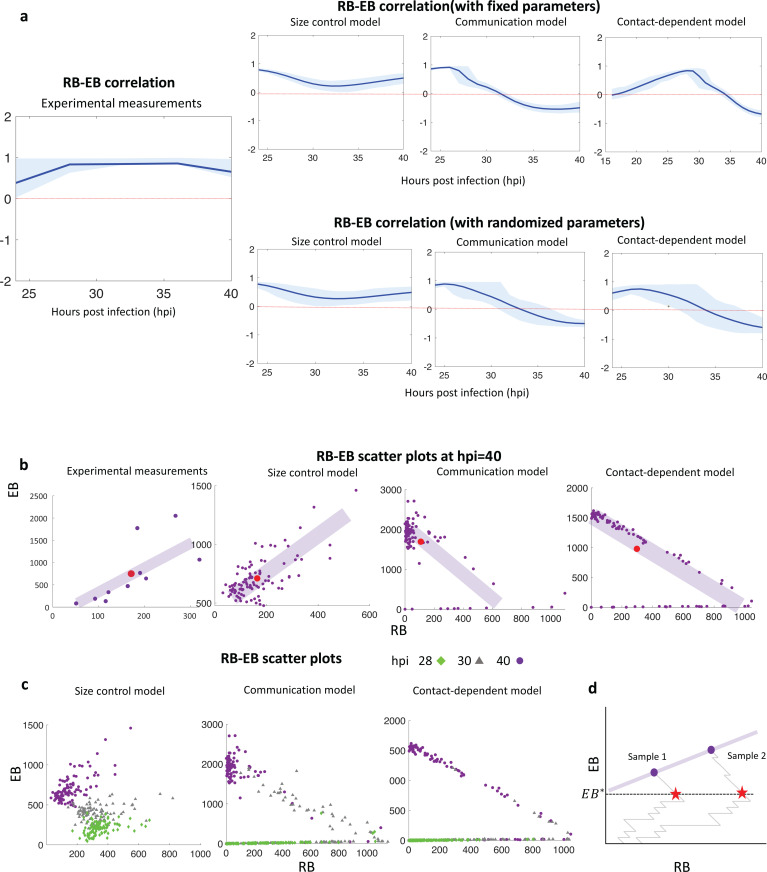
a. The time evolution of the correlation between RB and EB numbers for the experimental data and each model with fixed parameter sets (top) and with randomized parameter sets (bottom). The error bands (shaded areas) are computed with 100 data sets computed with the fixed parameter (top) and by randomly perturbing the given parameters (bottom). These 80%-error bands provide uncertainty analyses for system-intrinsic noise and parameter perturbations, respectively. The solid lines are the mean of the 100 RB-EB correlations obtained with the data sets. b. The scatter plots of the experimental data and the three models at 40 hpi. c. The scatter plots of each model at at three time points (28, 32, and 40 hpi). The purple shaded bars schematically indicate the linear trends of the data points with positive/negative slopes. d. Two samples of the communication model at 40 hpi can be aligned on a positively-sloped straight line when the first sample trajectory, which hits the threshold with a lower amount of RBs, ends up with fewer EBs and RBs than the second sample trajectory. These events occurred with low probability (see Section E of S1 Text).

In the experimental data, we observed a positive correlation between the number of RBs and EBs at all time points after 24 hpi (Fig 5a). For example, in an analysis of individual inclusions at 40 hpi, the number of RBs and EBs roughly aligned on a straight line with a positive slope (Fig 5c, left). This positive correlation indicates that there is a consistent relationship between the number of RBs and EBs, such that when there are more RBs, there are also more EBs.

All three models showed a positive correlation between RB and EB numbers at time points prior to 24 hpi, when EBs are first detected experimentally. However, only the size control model maintained a positive correlation of RB and EB number from 12 to 40 hpi, similar to the experimental measurements (Fig 5a). In contrast, the RB/EB correlation turned negative for the communication model after 32 hpi, and for the contact-dependent model after 28 hpi. This difference at late times in the intracellular infection is even more striking in scatter plots for RB number vs EB number in multiple simulated inclusions at 40 hpi. Both the communication and contact-dependent models formed a straight line with a negative slope, unlike the experimental data and the size control model which had a positive slope (Fig 5b).

We next investigated if the communication and contact-dependent models share a common feature that could account for the negative correlation between RB and EB numbers at late times. For these comparisons, scatter plots for each model again provided valuable insights. Both models produced a gradual increase in RB and EB numbers until they reached a threshold number (given by EBs for the communication model (Fig 3c) and by the total number of chlamydiae for the contact-dependent model (Fig 4c)). After reaching the threshold, the number of EBs greatly increased and the number of RBs greatly decreased, consistent with a dramatic rise in RB-to-EB conversion (Fig 5c). For both models, therefore, the scatter plots of (RB,EB) aligned on a straight line with a negative slope, consistent with a negative correlation. Although there is a possibility that two sample points form a straight line with a positive slope (Fig 5d), this event occurs with small probability (see Section E of S1 Text). In short, these two models are each controlled by an extrinsic signal and produce a runaway increase in RB-to-EB conversion, leading to a negative correlation between RB and EB numbers at late times. In contrast, the size control model did not produce a dramatic increase in the number of EBs at late times and maintained a positive correlation between RB and EB numbers at all time points (Fig 5c). We note that the growth event $R_{k}\to R_{k+1}$ is important for the positive correlation as due to this event, individual RBs have more heterogeneous division onset times (Fig A in S1 Text).

### Monotonic EB coefficient of variation

We employed another statistical feature, the coefficient of variation of EB number, CV(EB(t)), at each time point, to further compare the three mathematical models. From the experimental data sets and the simulated time trajectories, CV(EB(t)) is empirically calculated as


$$\begin{array}{c} \hat{CV}\left(EB(t)\right)=\sqrt{\frac{s_{EB}^{2}(t)}{\overset{―}{EB}(t{)}^{2}}}=\sqrt{\frac{\frac{1}{N-1}\sum_{i}{\left(EB_{i}(t)-\overset{―}{EB}(t)\right)}^{2}}{\overset{―}{EB}(t{)}^{2}}.} \end{array}$$ (2)

**Fig 6:**
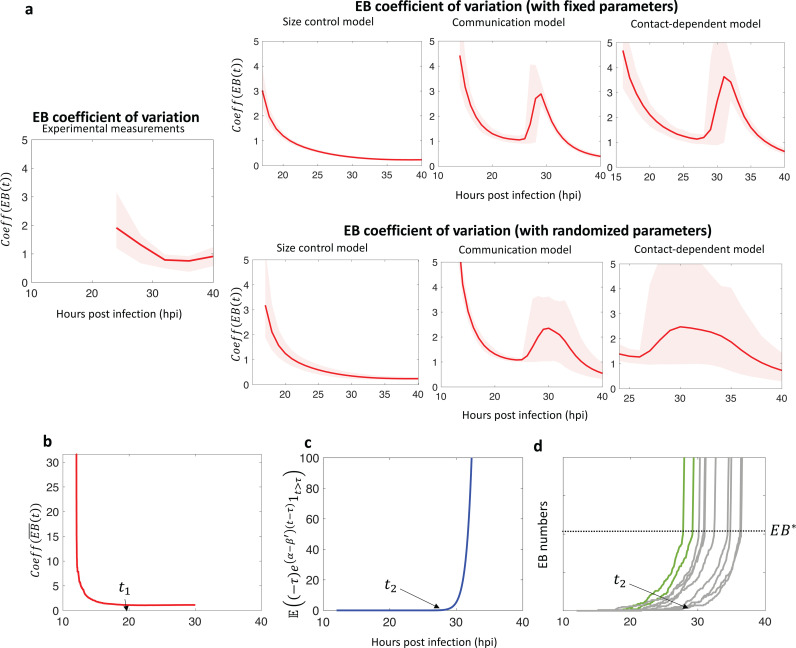
a. The graphs of $\hat{CV}(EB\left(t\right))$ for each model with fixed parameter sets (top) and with randomized parameter sets (bottom). The error bands are computed with 100 data sets computed with the fixed parameter (top) or by randomly perturbing the given parameters (bottom). The solid lines are the mean of the 100 EB coefficient of variation obtained with the data sets. b-c. The graphs of $\hat{CV}(\tilde{EB}\left(t)\right)$ and $𝔼\left(\left(t-\tau \right)e(\alpha -\beta \prime )\left(t-\tau \right)1_{t>\tau }\right)$ for the communication model with the parameters α=0.4, β=0.04 and β′=0.8. d. 10 sample trajectories of EB(t) of the communication model. Only two trajectories (green) passed the threshold by the designated time $t_{2}$.

This statistical quantity represents a scaled level of variability (noise) of a random variable. The statistics for the experimentally measured $\hat{CV}(EB(t))$ is undefined before 24 hpi, since no EBs have yet been produced, and it has a broadly decaying graph after 24 hpi (Fig 6a), except for one time point, 36hpi, when it slightly increases. The size control model produced a similarly shaped, monotonically decreasing graph for $\hat{CV}(EB(t))$ as seen with the experimental measurements. In contrast, the $\hat{CV}(EB(t))$ for the communication and contact-dependent models showed non-monotonic behavior around 28 hpi when RB-to-EB conversion was underway (Fig 6a). These disparities between the models were observed under a broad range of systems parameters and were not restricted to specific parameter choices (Fig 6a, bottom).

The communication model and the contact-dependent model share features that could explain their non-monotonic behavior in $\hat{CV}(EB(t))$. As illustrated in Fig 6c, these two models both produce a high level of RB-to-EB conversion around the threshold size, and the effect of small fluctuations in the time when EBs reach the threshold can be amplified to produce many more EBs, as illustrated with the green trajectories in Fig 6e. Hence the corresponding inclusions have much more EB variability around the average conversion onset time. Note that $\hat{CV}(EB(t))$ is still non-monotonic with an extrinsic signal modeled by a gradually increasing regulatory function rather than the step functions in Figs 3c or 4c (Fig B in S1 Text). With the size control model, however, conversion of an RB into an EB is intrinsically controlled, and not subject to fluctuation effects caused by an extrinsic signal, as shown in Fig 5c.

Using the properties of Poisson processes [25,26] we mathematically verified what we described in the previous paragraph. We used the communication model to illustrate our theoretic framework. An analysis for the contact-dependent and size control models can be done similarly. Recall that the communication model has two different phases before and after the EB threshold number is reached. When the threshold number of EBs is reached, the conversion rate dramatically changes from f(EB)=β to $f(EB)=\beta^{\prime }$ with $\beta^{\prime }\gg \beta$. Furthermore, to realize the bang-bang control, it necessarily holds that β<α and $\alpha <\beta^{\prime }$, where α is the division rate of RBs.

We expect a fluctuation of EB number at the conversion onset time $\tau =inf\left\{t>0:EB(t)=EB^{*}\right\}$ due to the dramatic change in the conversion rate (Fig 5c) that cause some outliers to show up as described in Fig 6d. Therefore, in order to study the non-monotonic behavior of $\hat{CV}(EB(t))$, we will select two time points $t_{1}<$ $t_{2}$, which are time points right before and after τ, such that $\hat{CV}(EB\left(t_{1}\right))$<$\hat{CV}(EB\left(t_{2}\right))$.

We first consider an extrinsic-signal-free process $\left(\tilde{RB}(t),\tilde{EB}(t)\right)$ that is the Markov chain associated with the reaction network shown in Fig 3c with f≡β. Note that before EB(t) reaches $EB^{*}$, both (RB(t),EB(t)) and $\left(\tilde{RB}(t),\tilde{EB}(t)\right)$have the same behavior. Since $\left(\tilde{RB}(t),\tilde{EB}(t)\right)$ has linear transition rates, we can find explicit forms of the variance and the mean of $\tilde{EB}(t).$Using the explicit forms, we further show that the coefficient variation of $\tilde{EB}$, $CV(\tilde{EB}(t))$, converges to a constant c exponentially fast with the rate $e^{-(\alpha -\beta )t}$ (Fig 6b). This will imply that if the conversion rate β is small enough, $CV(\tilde{EB}(t))$ is nearly c for both $t=t_{1}$ and $t=t_{2}$ as long as they are not too small. See more details in Section F of S1 Text.

Now we select $t_{1}$ so that $P(t_{1}<\tau )$ is large. Hence EB(t) behaves almost identically as $\tilde{EB}(t)$ around $t=t_{1}$. By the exponentially fast decay of $CV(\tilde{EB}(t))$ over time, we expect that $CV(EB\left(t_{1}\right)\approx CV(\tilde{EB}\left(t_{1}\right))\approx CV(\tilde{EB}\left(t_{2}\right))\approx c=\lim_{t\to \infty }CV(\tilde{EB}(t))$. Furthermore, we show that if $\beta <\alpha <\beta^{\prime }$ and if we can select $t_{2}$ as $𝔼\left(\left(t_{2}-\tau \right)e^{(\alpha -\beta^{\prime })\left(t_{2}-\tau \right)}1_{t_{2}>\tau }\right)$ is small, then $CV\left(EB\left(t_{2}\right)\right)=CV\left(\overset{―}{EB}\left(t_{2}\right)\right)+c^{\prime }$ for some constant $c^{\prime }$. Here $c^{\prime }$ is positive if $\beta <\alpha <\beta^{\prime }$ and if $EB^{*}$ is not too small. This completes our goal $CV(EB(t_{1}))$<$CV(EB(t_{2}))$ (See Section F of S1 Text for more details). The choice of such a $t_{2}$ is plausible as we selected $\beta^{\prime }>\alpha$ so that the term $e^{(\alpha -\beta^{\prime })\left(t_{2}-\tau \right)}$ is not big (Fig 6c). Furthermore, if the division rate α is sufficiently large compared with the small conversion rate β, the convergence of CV(EB(t)) will occur earlier so that we can choose small enough $t_{1}$. This allows us to pick the two time points satisfying $t_{2}>t_{1}$.

## Alternative parameter sets

One concern that might be raised regarding the communication model and the contact-dependent model is that while the fit is not ideal to experimental data with the given set of parameters, another set of parameters might provide a better fit. We explored this question in detail by considering over 100,000 parameter sets looking for a basic fit to the experimental data. The desired criteria include 1) a rough fit to the population graphs with an initial average RB growth, followed by a decrease in RB and increase in EB, 2) a positive correlation at all times between RB and EB, and 3) no spikes of EB noise. As it turns out, not a single one of the parameter sets sampled for these two models is able to reproduce such basic conditions. Although this might come as a surprise, it is a natural result considering the intuitive reasoning why these models are not a good qualitative fit to the data, as described in detail above. Moreover, we show that given a parameter set of either the communication model or the contact dependent model satisfying criterion 1), then necessarily criterion 2) must fail, and criterion 3) must fail.

On the other hand, a similar approach was carried out for the size control model itself. Using the same three basic criteria, the parameters of this model were broadly varied over multiple orders of magnitude and tested forbro qualitative fit. Unlike the two other models, the size control model satisfied the criteria for 10% of the parameter sets. This shows that is broadly possible for this model to reproduce the experimental data.

See the Section G of S1 Text for additional details on these results, including the choice of the parameter sets, the specific expected criteria, and the ranges for each parameter.

## Discussion

The conversion of the reproductive RB into the infectious EB is a critical step in the intracellular *Chlamydia* infection that only takes place inside a mammalian host cell. It is important to regulate the switch from RB replication to RB-to-EB conversion to maximize EB production [14] Understanding the mechanism that controls conversion is likely to lead to important insights into the dynamics of *Chlamydia* infections, as well as providing potential new targets for anti-chlamydial drugs and other treatments. This point was made clear by groups such as Wilson et al. [6], Chiarelli et al. [12], Omsland et al. [10] who have tried to determine the mechanism controlling RB-to-EB conversion.

This work has a number of strengths over previous published modeling studies of the intracellular *Chlamydia* infection: i) using a detailed experimental dataset with exact numbers of each chlamydial developmental form, ii) incorporating stochasticity in the mathematical models, iii) identifying statistical properties that are experimentally measurable and can distinguish between models, iv) providing an in-depth mathematical analysis with partial proofs of our conclusions, v) systematically varying parameter values in each model to further support our conclusions, vi) combining these approaches to compare specific models of RB-to-EB conversion. This study advances this field of inquiry by comparing three specific mechanisms of conversion from the literature, and by broadly showing via extensive simulations and mathematical analysis that only intrinsic mechanisms are consistent with available stochastic information.

The work by Chiarelli et al. [12] studied two generic, deterministic models of Chlamydia conversion, one intrinsic and one extrinsic. It found that the intrinsic model was more likely to be consistent with the fact that EB production was not experimentally influenced by increased multiplicity of infection or by superinfection. We have significantly expanded on this work by looking at specific regulatory mechanisms from the existing literature such as the contact-dependent conversion [6] or the size control model [11], and by including extensive simulations with many parameter sets, mathematical analysis of each system, and inclusion of stochastic effects in our mathematical models. Stochastic information is readily available in experimental data, and it is also an integral part of the infection, such as the asynchrony in conversion inside each inclusion. While Chiarelli et al. have recently implemented stochastic agent-based models [13], they did so mostly to study the relevance of asymmetric division, not to distinguish between different conversion mechanisms. Notice also that while the previous work by Chiarelli et al. used an external signal, this signal is consumed like a nutrient, while ours is modeled as a quorum-sensing signal. Nevertheless, our analysis provides evidence that even if the signal was consumed, the mechanism is not likely to reproduce the stochastic experimental evidence.

For each of the three mechanisms considered, we developed a mathematical model to identify parameters that could reproduce the experimentally measured growth curves for RBs and EBs. We also identified two stochastic properties of the experimental data that allowed us to distinguish between the three models. The first property is a positive correlation between RB and EB numbers at late time points, which was present in the experimental data and reproduced by the size control model, but not the communication and contact-dependent models, which both had a negative correlation. The second property is the EB coefficient of variation, which monotonically decreased as a function of time in the experimental data and the size control model but increased around the time of RB-to-EB conversion for both the communication and contact-dependent models.

These statistical differences can be explained by the dynamics of RB-to-EB conversion for the different regulatory models. The size control model regulates conversion through an *intrinsic* property of each RB (its size) that does not depend on extrinsic factors. Variability in early dynamics can lead some inclusions to have many RBs and to produce many EBs, while other inclusions can have limited numbers of both RBs and EBs. In this way, a positive correlation between the number of RBs and EBs within an inclusion is maintained at all times, which is consistent with the experimental data.

In contrast, both the communication model and the contact-dependent model have a conversion trigger that is *extrinsic* to the individual RB. For the communication model, the putative signal is produced by EBs, and the conversion threshold is thus dependent on the size of the chlamydial population. For the contact-dependent model, the conversion threshold is dependent on inclusion size because a larger inclusion decreases the contact area between the RB and the inclusion membrane, which may promote RB detachment and conversion. For both models, the external signal functions as an inclusion-wide trigger for conversion, which leads to a large increase in the number of EBs and a concomitant decrease in the number of RBs. However, due to stochastic variability in the time when the size threshold is reached, some inclusions will have more RBs and fewer EBs, and other inclusions will have fewer RBs and more EBs at a given time point. This effect gives rise to a range of inclusions at a fixed time point that lie on a line with a negative slope, as illustrated in Fig 6c. Thus, our two models that depend on an extrinsic conversion signal produced a negative correlation between RB and EB numbers at late times, which is inconsistent with the experimental data. These observations are backed by mathematical analysis in our work, and by significantly varying the parameters of each of the two models over 100,000 times. Not a single parameter set satisfied three basic, experimentally observed criteria. This provides evidence that the contact-dependent and communication model are structurally unable to reproduce the known data about the Chlamydia developmental cycle.

We propose that the different effects of extrinsic and intrinsic signals can be generalized beyond the three models that we compared in this study. Any *Chlamydia* developmental model controlled by an extrinsic signal will likely exhibit similar statistical features of runaway RB-to-EB conversion if the extrinsic signal affects all RBs. In contrast, an RB-intrinsic signal, by its very nature, acts at the level of an individual RB, and each RB converts independently into an EB. As a result, the relationship between RB and EB number is preserved, and an inclusion with more RBs will produce proportionally more EBs, maintaining a positive RB/EB correlation. Also, with an intrinsic signal, the timing of conversion will not be synchronized, and a few outliers in the number of EBs will not significantly increase the variance of the system, yielding a broadly monotonic behavior for the EB coefficient of variation.

Our paper has two novel features compared to previous mathematical modeling of the chlamydial developmental cycle. Firstly, we used chemical reaction networks and the associated Markov chains to provide unified mathematical frameworks so that we could compare the three models. In particular, the models share the same mathematical settings; each reaction fires with exponential holding times, and the reaction intensities are commonly defined with mass-action kinetics for the three models. Under these common settings, we only used different architectures of the reaction networks to characterize the three mechanistic models. The [11], study modeled the size control mechanism by using RB-division times that followed Gamma distributions. For our purposes, we did not use this approach because it could not be applied to the two other models. That previous model also did not take into account statistical features such as the correlation and the coefficient of variation. A second novelty is that the models can capture the details of their respective control mechanism and yet are still simple enough for analyzing their statistical features heuristically and theoretically. The Markov models we proposed are not coarse-grained, as they reproduce the qualitative behavior of the experimentally measured chlamydial growth curve. On the other hand, due to their simplicity (e.g., only a few reactions were required to describe the communication model and the contact-dependent model), we were able to understand heuristically and theoretically how and why the models have different statistical features.

In summary, we have modeled three potential mechanisms for controlling RB-to-EB conversion during the chlamydial developmental cycle inside an infected host cell. All three models were able to recapitulate the growth curves of RBs and EBs during the stages of RB replication and RB-to-EB conversion. However, we identified two statistical properties, the correlation between the number of RBs and EBs at late times, and the coefficient of variation of EB numbers, that allowed us to distinguish between the models. Of our three models, only the size control model resembled the experimental data in having both a positive correlation between RB and EB number at late times and a monotonic time course for the coefficient of variation of EB number. Our analysis suggests that these statistical shortcomings of the communication and contact-dependent models are due to their reliance on an extrinsic signal that may lead to the runaway production of EBs at late times. Thus, this study highlights the advantage of using an intrinsic signal to control RB-to-EB conversion and provides support for the size control model as its regulatory mechanism. RB-to-EB conversion is critical because only EBs can spread the intracellular infection to a new host cell. and this essential step may be an attractive therapeutic target for novel anti-*Chlamydia* antibiotics to combat this highly prevalent pathogen.

## Methods

Reaction networks: In this paper, we used reaction networks to model the three different mechanisms for the chlamydia developmental cycle. Reaction networks are graphical configurations that represent interactions between chemical species such as


$$\begin{array}{c} X_{1}+X_{2}\overset{\lambda_{1}(X)}{\to }X_{3},X_{1}\overset{\lambda_{2}(X)}{\to }X_{2},X_{2}\overset{\lambda_{3}(X)}{\to }X_{1}. \end{array}$$ (3)

The nodes such as $X_{1}+X_{2}$ and $X_{3}$ are called complexes, and the oriented edges between two complexes such as $X_{1}+X_{2}\to X_{3}$ are called reactions. The variables constituting a reaction network such as $X_{1},X_{2}$ and $X_{3}$ are called species. In general, reactions and complexes in a reaction network can be represented as


$$\begin{array}{c} \sum_{i=1}^{d}{y_{i}X}_{i}\overset{\lambda_{y\to y^{\prime }}(X)}{\to }\sum_{i=1}^{d}{{y^{\prime }}_{i}X}_{i}, \end{array}$$ (4)

where $y_{i}$ and $y_{i}^{\prime }$ for i=1,2,…,d are non-negative integers. The vector ${\left(y_{1}^{\prime },\ldots ,y_{d}^{\prime }\right)}^{⊤}-{\left(y_{1},\ldots ,y_{d}\right)}^{⊤}$ is called a reaction vector or a stoichiometric vector. Each coordinate of this vector gives the net gain of ith species through a reaction. The function $\lambda_{y\to y^{\prime }}(X)$ indicates the intensity of the reaction as a function of the current state of the system X, which is often defined under mass-action kinetics. Additional information regarding deterministic reaction networks can be found for instance in [18]. Additional information on modeling stochastic processes associated with reaction networks is also included in S1 Text.

Parameter fitting with the simulated annealing algorithm: For each model, we fit the parameters using the simulated annealing algorithm with the loss function $L(RB,EB)=\sum {\mathrm{\backslash nolimits}}_{i=1}^{8}({\left|RB\left(t_{i}\right)-\hat{RB}\left(t_{i}\right)\right|}^{2}+0.1{\left|EB\left(t_{i}\right)-\hat{EB}\left(t_{i}\right)\right|}^{2}$, where (RB,EB) and $\left(\hat{RB},\hat{EB}\right)$ are the time trajectories of RB and EB numbers obtained with the model simulation and the experimental measurements, respectively. The times $t_{i}$ used are the 8 time points 12,16,18,20,24,28,32,36 and 40 hpi. As the bang-bang behavior of RB is a key feature of the model, we weighted the error ${\left|EB\left(t_{i}\right)-\hat{EB}\left(t_{i}\right)\right|}^{2}$ with 0.1 in the loss function. From the initial parameters, we search randomly a new set of parameters, each of which is searched randomly as $x_{new}=x+xU_{x}$, where $U_{x}\sim Uniform(-0.2,0.2).$ The precise psedo-algorithm is provided in Section G of S1 Text.

## Supporting information

S1 Text Supplementary text, including an introduction to stochastically modeled reaction networks as well as an in-depth study of each of the three models, the RB-to-EB correlation, nonmonotonic behavior of the noise, and parameter fitting. Also included here are Fig A: mean (left) and RB-EB correlation (middle) time trajectories of the size control model; Fig B: bang-bang behavior of the communication and the contact-dependent models under various choices of parameters (top), the corresponding RB-EB correlations (middle) and the EB coefficients of variation (bottom); Fig C: time evolutions of the statistical features of the communication model with a smooth extrinsic signal.
(PDF)

S1 Table Data from Lee et al. including for each inclusion its corresponding time post infection (hpi), and the count of all primary (RB + DB) and secondary (IB + EB) forms. (PDF)
