# Supplementary material for: Statistical analysis supports the size control mechanism of Chlamydia development
Source: PLoS Comput Biol. 2025 Jul 14;21(7):e1013227. doi: 10.1371/journal.pcbi.1013227 (PMC12279117; doi:10.1371/journal.pcbi.1013227)
Supplement: S1 Text — Also included here are Fig A: mean (left) and RB-EB correlation (middle) time trajectories of the size control model; Fig B: bang-bang behavior of the communication and the contact-dependent models under various choices of parameters (top), the corresponding RB-EB correlations (middle) and the EB coefficients of variation (bottom); Fig C: time evolutions of the statistical features of the communication model with a smooth extrinsic signal. (PDF) [file pcbi.1013227.s001.pdf]

Supplementary Material: Statistical analysis supports size control mechanism of Chlamydia development

Jinsu Kim, Christine Sütterlin, Ming Tan, and German Enciso

Contents

S.A Stochastically modeled reaction networks **1**

S.B Size control model **2**

S.C Communication model **3**
- S.C.1 The case of RB-derived signals 4
- S.C.2 The case of EB-derived negative signals 5
- S.C.3 Moments equations of the communication model 6

S.D Contact-dependent model **7**

S.E Correlation coefficient between RB and EB **8**
- S.E.1 Positive correlation coefficient for the size-control model 8
- S.E.2 Negative correlation coefficient for the communication model 9

S.F Non-monotonic behavior of the EB coefficient for the communication model **10**

S.G Choice of system parameters **13**
- S.G.1 Robustness of the statistical features to parameters that induce the bang-bang behavior 14
- SG.2 Pseudo codes for the simulated annealing algorithm 16

S.H Statistical features of the communication model with EB-derived positive feedback modeled by a smooth signal, $f(EB)$. **16**

S.A Stochastically modeled reaction networks

See Method in the main text for the basic terminologies about reaction networks. Assuming the space of a reaction system is well-mixed, a system of ordinary differential equations is often employed to model the concentration of species involved in a reaction network, especially when the abundance of the copy numbers of species is sufficiently large. However, when the system abundance is small, we often use continuous-time Markov chains to model the counts of species. For

a given reaction network, let $\mathcal{R}$ denote the set of reactions $\{y \to y' \in \mathcal{R}\}$. Then a Markov chain $X(t) \in \mathbb{Z}^d_{\geq 0}$ associated with the reaction network has the following transition rates [2]:

$$P(X(t+\Delta t) = z | X(t) = x) = \sum_{\{y\to y' \in \mathcal{R}: y'-y=z-x\}} \lambda_{y\to y'}(x)\Delta t + o(\Delta t), \tag{1}$$

where $\lambda_{y\to y'}(x)$ is called the *intensity* of the reaction $y \to y'$.

One of the most common choices of the intensities is *mass action* that holds

$$\lambda_{y\to y'}(x) = \kappa_{y\to y'} \prod_{i=1}^{d} x_i(x_i - 1)\cdots(x_i - y_i + 1)\mathbb{1}_{\{x_i \geq y_i\}},$$

where $\kappa_{y\to y'}$ is a positive constant, and we call it a *rate constant*. Typically, we incorporate the rate constant into the reaction network by placing it on top of the reaction arrow as $y \xrightarrow{\kappa_{y\to y'}} y'$ when the reaction intensity follows mass-action kinetics. However as some reactions in our models do not obey mass action, we denote the reactions as $y \xrightarrow{\lambda_{y\to y'}(X)} y$, where $\lambda_{y\to y'}$ is the intensity defined with a general function of X. For instance, for $RB \xrightarrow{\lambda_{RB\to EB}(X)} EB$ in the communication model, the reaction intensity for this conversion is $\lambda_{RB\to EB}(RB, EB) = f(EB)RB$ with a step function f.

Finally, with the transition rates given at (1), the chemical master equation, which is a system of ordinary differential equations for the probability density $p(x,t) = P(X(t) = x)$, is

$$\frac{d}{dt}p(x,t) = \sum_{y\to y' \in \mathcal{R}} \lambda_{y\to y'}(x - y' + y)p(x - y' + y, t) - \sum_{y\to y' \in \mathcal{R}} \lambda_{y\to y'}(x)p(x,t).$$

S.B Size control model

As proposed in [6], RBs are initially as large as about size $1\mu m^3$ and reduce their size progressively until their size is around $0.06\mu m^3$ and then convert to EB. To model the size control hypothesis, we first subdivide the size interval $[0, 06, 1]$ into M different size bins. Then the RBs in a single inclusion are assigned to one of the size bins according to their size. Then we denote by $RB_k(t)$ the number of RBs belonging to the kth size bin, where RB_0 represents the smallest size bin and RB_M represents the biggest size bin.

The size control model is a stochastic process

$$X(t) = (RB_0(t), RB_1(t), \dots, RB_M(t), EB(t))^\top \in \mathbb{Z}^{M+2}_{\geq 0}$$

that is a continuous-time Markov chain. This Markov chain can be associated with the following reaction network:

$$\begin{aligned} &RB_k \xrightarrow{\alpha RB_k} 2RB_{k-1} \quad \text{for } k = 1, 2, \dots, M \\ &RB_k \xrightarrow{\gamma RB_k} RB_{k+1} \quad \text{for } k = 1, 2, \dots, M-1 \\ &RB_0 \xrightarrow{\beta RB_0} EB. \end{aligned} \tag{2}$$

Then the transition rates of this Markov chain can be modeled as a mass action system as shown in (2), and hence the chemical master equation for $p(x,t) = P(X(t) = x)$ is

$$\begin{aligned}\frac{d}{dt}p(x,t) = \alpha \sum_{i=2}^{M+1} p(x - 2e_{i-1} + e_i, t)(x_i + 1) \\ + \gamma \sum_{i=2}^{M} p(x - e_{i+1} + e_i, t)(x_i - 1) \\ + \beta p(x - e_{M+2} + e_1, t)(x_1 + 1) \\ - \left(\alpha \sum_{i=2}^{M+1} x_i + \gamma \sum_{i=2}^{M} x_i + \beta x_1 \right) p(x,t),\end{aligned} \tag{3}$$

where $e_i \in \mathbb{Z}^d$ is the elementary vector whose ith component is 1 and the other components are zero.

The growth reaction is crucial as it leads to an uneven onset time for individual RB conversion. As shown in Figure A, when $\gamma = 0$, the correlation between RB and EB becomes negative at later time points, while the average curve accurately reflects the bang-bang behavior. The scatter plots indicate that the conversion of RBs to EBs occurs in a timely and homogeneous manner.

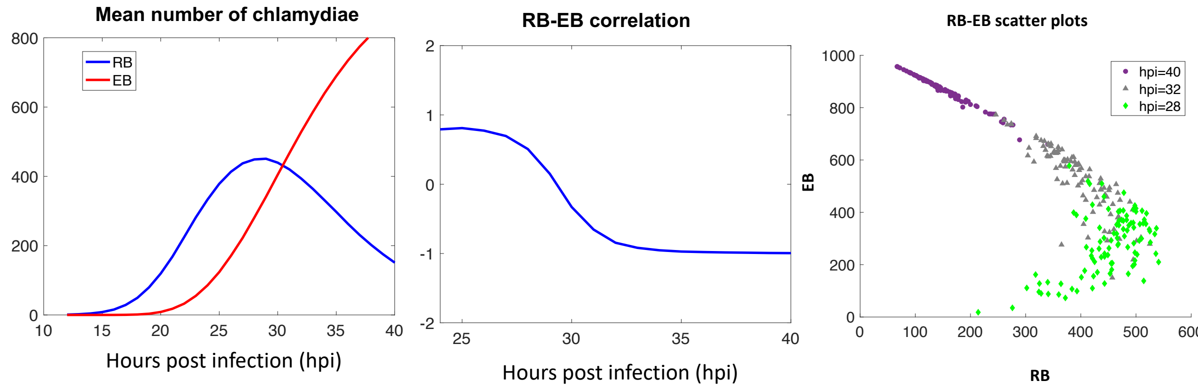

Figure A: Mean (left) and RB-EB correlation (middle) time trajectories of the size control model with γ =0. Ahe Acatter Alot Ahows Ahe ABs Aonvert Ao ABs At Aimilar Aimes Aright).

S.C Communication model

The communication model assumes that the rate of RB-to-EB conversion is controlled by signals sent from either RBs or EBs. The signal can be also either positive or negative. Hence, we can think of two different reaction networks such as

$$RB \xrightarrow{\alpha RB} 2RB, \quad RB \xrightarrow{f(EB)RB} EB, \tag{4}$$

for which the conversion is controlled by signals of EBs, and

$$RB \xrightarrow{\alpha RB} 2RB, \quad RB \xrightarrow{f(RB)RB} EB, \tag{5}$$

for which the conversion is controlled by signals of RBs. Furthermore, if the function f in (4) and (5) is increasing, the signal is positive, and if it is decreasing, the signal is negative on the conversion.

In the main text, we used the model in (4) with an increasing function $f(EB)$. In the following subsections, we show how the other models are ruled out due to the lack of reproducibility of the experimental data.

S.C.1 The case of RB-derived signals

In this section, we tested the plausibility of the scenario about the mechanism with signals of RBs on the RB-to-EB conversion, which can be modeled with the reaction network in (5). We will conclude that if $f(RB)$ is either a step function or a linear function, the mean $E(RB(t))$ fails to reproduce the mean measured with the experimental data whose graph is shown in Figure 1d.

We first assume that $f(RB)$ is a step function as suggested in the main text. That is,

$$f(RB) = \begin{cases} \beta' & \text{if } RB > RB^*, \\ \beta & \text{if } RB \le RB^*, \end{cases}$$

where RB^* is a constant that gives the threshold for the phase transition of the RB-to-EB conversion. If $\beta' < \beta$, RBs more slowly convert to an EB after it hits the threshold RB^*, which is not consistent with the behavior of the bang-bang control. Thus we expect that $\beta' > \beta$.

The setting $\beta' > \beta$ causes $RB(t)$ to oscillate around the threshold because of the following reasons. For a sample trajectory $RB(t)$, if it exceeds RB^* at $t = \tau_1$, it will degrade strongly as $\beta' > \beta$. Then it will hit the threshold again at some time $t = \tau_2$. Then $RB(t)$ will increase due to less conversion (i.e. $\beta' > \beta$) and hence hit again the threshold at some time $t = \tau_3$, and repeat the processes above. On average, this will lead a sustained mean dynamics of $RB(t)$ around RB^* (Figure 3a iii)) that is not observed in the experimental measurements (Figure 1d).

Next, we assume that f is a smooth function of the RB number. We use the mean and variance of $RB(t)$ to show that this mechanism fails to reproduce the bang-bang control in the mean dynamics of the RB and EB numbers (Figure 1d). The main idea is to use the fact that autonomous ordinary differential equations cannot have non-monotonic dynamics over time.

Note that in the system (5) modeled with the RB signals, the conversion rate is

$$\lambda_{RB\to EB}(RB, EB) = f(RB)RB := F(RB),$$

where f is either increasing (positive signal) or decreasing (negative signal) as a function of the RB numbers. By assuming the signal f is almost linearly growing with RB, we further suppose that the Taylor expansion of F can be approximated with up to its second-order term, as $F(RB) \approx aRB + bRB^2$ for some constants a and b. Using the random time representation [1, 2, 4], then, the RB number in the stochastic modeling of the communication model is

$$RB(t) = RB(0) - Y_1\left(\int_0^t F(RB(s))ds\right) + Y_2\left(\int_0^t \alpha RB(s)ds\right), \qquad (6)$$

where αRB is the division rate of RB, and the Y_1 and Y_2 are independent unit Poisson processes. The master equation of $RB(t)$ is, for $P(RB(t) = x) = p(x,t)$,

$$\frac{d}{dt}p(x,t) = F(x+1)p(x+1,t) + \alpha(x-1)p(x-1,t) - (F(x) + \alpha x)\,p(x,t). \qquad (7)$$

Using either (6) or (7), we can derive the ordinary differential equation of the average RB numbers as

$$\begin{aligned}\frac{d}{dt}\mathbb{E}(RB(t)) &= -\mathbb{E}(F(RB(t))) + \alpha\mathbb{E}(RB(t)) \\ &\approx (\alpha - a)\mathbb{E}(RB(t)) - b\mathbb{E}(RB(t)^2) \\ &= (\alpha - a)\mathbb{E}(RB(t)) + b\mathbb{E}(RB(t))^2 - bVar(RB(t)),\end{aligned} \tag{8}$$

where we used the quadratic approximation $F(x) \approx ax + bx^2$ for the second inequality above. As shown in Figure 1d, the experimentally measured average RB numbers display non-monotonic behavior having a peak at $t = 32$. Hence for some $\delta > 0$, $\mathbb{E}(RB(32 - \delta)) = \mathbb{E}(RB(32 + \delta))$. If we model the RB numbers (or concentration) deterministically, then $Var(RB(t))$ is identically zero in (8) implying the corresponding ordinary differential equation for $\mathbb{E}(RB)$ is autonomous. Hence it is impossible that $\mathbb{E}(RB(32 - \delta)) = \mathbb{E}(RB(32 + \delta))$ while the derivatives of $\mathbb{E}(RB(t))$ are distinct at $t = 32 - \delta$ and $t = 32 + \delta$. In the stochastic modeling, although $Var(RB(t)) > 0$, we know that the experimentally measured coefficient of variation of RB is almost a constant as displayed in Figure 6a after $t = 30$. This implies that $Var(RB(t)) \approx c\mathbb{E}(RB(t))^2$ for some $c > 0$ if $t \geq 30$. This observation makes the ordinary differential equation of $\mathbb{E}(RB)$ almost autonomous as

$$\frac{d}{dt}\mathbb{E}(RB(t)) \approx (\alpha - a)\mathbb{E}(RB(t)) + b(1 - c)\mathbb{E}(RB(t))^2 \quad \text{for } t \geq 30,$$

concluding that $\mathbb{E}(RB)$ follows an autonomous differential equation. Hence it cannot have the same value at different time points with different derivatives. Therefore for the stochastic modeling of the communication model with the RB signal on the RB-to-EB conversion rate, the non-monotonic behavior of $\mathbb{E}(RB)$, which is experimentally verified, cannot be reproduced.

S.C.2 The case of EB-derived negative signals

One can expect that if the number of EB negatively affects the conversion rate (i.e. $f(EB)$ is a decreasing function), then more RB's remain at later time points, which in turn leads to a higher conversion rate. However, mathematically this setting leads to an exponential growth of RBs at later time points as opposed to the experimental measurements.

To show this, for the Markov chain $X(t) = (RB(t), EB(t))^\top$ associated with (4), we set $\beta' < \beta$ for

$$f(EB) = \begin{cases} \beta' & \text{if } EB > EB^*, \\ \beta & \text{if } EB \leq EB^*, \end{cases} \tag{9}$$

with some threshold EB^*. Let $\tau = \inf\{t > 0 : EB(t) = EB^*\}$. For large t, we have by the differential equation of $\mathbb{E}_{X(\tau)}(RB(t))$ in (14) that

$$\mathbb{E}(RB(t)) \approx \mathbb{E}(RB(t)\mathbb{1}_{t\geq\tau}) = \mathbb{E}(\mathbb{E}_{X(\tau)}(RB(t-\tau))\mathbb{1}_{t\geq\tau}) = \mathbb{E}(RB(\tau))e^{(\alpha-\beta')t}\mathbb{E}(e^{-(\alpha-\beta')\tau}\mathbb{1}_{t\geq\tau})$$

This derivation is shown with more details in Section S.F. Hence if $\alpha > \beta'$, it is impossible for RB to decay at later time points. If $\alpha < \beta' < \beta$, the division rate is less than the conversion rates. Hence RB cannot grow initially as opposed to the experimental measurements (Figure 1d).

S.C.3 Moments equations of the communication model

In this section, we compute the moments of the communication model. The moment equations will be critically used in the later section to validate the negative correlation between RB and EB in later time points (Figure 5). Also, those are used to study the non-monotonic behavior of the time evolution of $CV(EB)$ (Figure 6).

Importantly, this model has two phases: before and after the threshold EB^* is reached. Let $\tau = \inf\{t \geq 0 : EB(t) = EB^*\}$ be the stopping time when the threshold is reached. Then we can decompose the mean number of RBs as

$$\mathbb{E}(RB^k(t)) = \mathbb{E}(RB^k(t)\mathbb{1}_{t<\tau}) + \mathbb{E}(RB^k(t)\mathbb{1}_{t\geq\tau}), \text{ and} \tag{10}$$

$$\mathbb{E}(EB^k(t)) = \mathbb{E}(EB^k(t)\mathbb{1}_{t<\tau}) + \mathbb{E}(EB^k(t)\mathbb{1}_{t\geq\tau}), \tag{11}$$

for each k.

For the first phase (i.e. $t < \tau$), we have

$$(RB(t), EB(t)) = (\widetilde{RB}(t), \widetilde{EB}(t)) \quad \text{in distribution given } t < \tau,$$

where $(\widetilde{RB}(t), \widetilde{EB}(t))$ is the Markov chain associated with

$$\widetilde{RB} \xrightarrow{\alpha\widetilde{RB}} 2\widetilde{RB}, \quad \widetilde{RB} \xrightarrow{\beta\widetilde{RB}} \widetilde{EB}, \tag{12}$$

which is introduced in the main text. Hence $(\widetilde{RB}(t), \widetilde{EB}(t))$ can represent the behavior of $\mathbb{E}(RB^k(t)\mathbb{1}_{t<\tau})$ and $\mathbb{E}(EB^k(t)\mathbb{1}_{t<\tau})$ when t is small. Note that $(\widetilde{RB}, \widetilde{EB})$ follows a linear system (i.e. the reactions in (12) are unimolecular or in other words, the reaction intensities are linear). In this case, there are a variety of ways of deriving moment equations. We use a Markov generator $\mathcal{A}$, which is defined for $(\widetilde{RB}, \widetilde{EB})$ as

$$\mathcal{A}V(x, y) = \alpha x(V(x + 1, y) - V(x, y)) + \beta x(V(x - 1, y + 1) - V(x, y)),$$

for a test function V. With test functions $V(x, y) = x, y, xy, x^2$ and y^2, Dynkin's formula [3] yields that

$$\begin{aligned}
&\mathbb{E}(\widetilde{RB}(t)) = \widetilde{RB}(0) + (\alpha - \beta)\int_0^t \mathbb{E}(\widetilde{RB}(s))ds,\\
&\mathbb{E}(\widetilde{EB}(t)) = \widetilde{EB}(0) + \beta\int_0^t \mathbb{E}(\widetilde{RB}(s))ds,\\
&\mathbb{E}(\widetilde{RB}(t)\widetilde{EB}(t)) = \widetilde{RB}(0)\widetilde{EB}(0) + (\alpha - \beta)\int_0^t \mathbb{E}(\widetilde{RB}(s)\widetilde{EB}(s))ds\\
&\qquad\qquad + \beta\int_0^t \mathbb{E}(\widetilde{RB}^2(s) - \widetilde{RB}(s))ds,\\
&\mathbb{E}(\widetilde{RB}^2(t)) = \widetilde{RB}^2(0) + 2(\alpha - \beta)\int_0^t \mathbb{E}(\widetilde{RB}^2(s))ds + (\alpha + \beta)\int_0^t \mathbb{E}(\widetilde{RB}(s))ds, \text{ and}\\
&\mathbb{E}(\widetilde{EB}^2(t)) = \widetilde{EB}^2(0) + 2\beta\int_0^t \mathbb{E}(\widetilde{RB}(s)\widetilde{EB}(s))ds + \beta\int_0^t \mathbb{E}(\widetilde{RB}(s))ds.
\end{aligned} \tag{13}$$

For the second phase, the moment equations are derived using conditional expectations. For example, by using the strong Markov property, we have

$$\mathbb{E}(RB(t)\mathbb{1}_{t>\tau}) = \mathbb{E}(\mathbb{E}(RB(t)|\mathcal{F}_\tau)\mathbb{1}_{t>\tau}) = \mathbb{E}(\mathbb{E}_{X(\tau)}(RB(t-\tau))\mathbb{1}_{t>\tau}).$$

Then we can derive moment equations for such conditional expectations in the same way we used for $(\widetilde{RB}, \widetilde{EB})$. Then we have

$$\begin{aligned}
&\mathbb{E}_{X(\tau)}(RB(t)) = RB(\tau) + (\alpha - \beta)\int_0^t \mathbb{E}_{X(\tau)}(RB(s))ds,\\
&\mathbb{E}_{X(\tau)}(EB(t)) = EB(\tau) + \beta\int_0^t \mathbb{E}_{X(\tau)}(RB(s))ds,\\
&\mathbb{E}_{X(\tau)}(RB(t)EB(t)) = RB(\tau)EB(\tau) + (\alpha - \beta)\int_0^t \mathbb{E}_{X(\tau)}(RB(s)EB(s))ds\\
&\qquad\qquad + \beta\int_0^t \mathbb{E}_\tau(RB^2(s) - RB(s))ds\\
&\mathbb{E}_{X(\tau)}(RB^2(t)) = RB^2(\tau) + 2(\alpha - \beta)\int_0^t \mathbb{E}_{X(\tau)}(RB^2(s))ds + (\alpha + \beta)\int_0^t \mathbb{E}_{X(\tau)}(RB(s))ds,\\
&\mathbb{E}_{X(\tau)}(EB^2(t)) = EB^2(\tau) + 2\beta\int_0^t \mathbb{E}_{X(\tau)}(RB(s)EB(s))ds + \beta\int_0^t \mathbb{E}_{X(\tau)}(RB(s))ds.
\end{aligned} \tag{14}$$

S.D Contact-dependent model

Under the contact-dependent hypothesis, the location of individual RBs decide the conversion rate: the RBs in the interior of the inclusion have a high change of converting to an EB while the RBs on the inclusion membrane may not. In this regard, an agent-based model could describe the RB-to-EB conversion mechanisms under the contact-dependent hypothesis, where each agent diffuses as a Brownian particle. This type of spatial-temporal stochastic processes are often employed for cellular biology systems and biophysical problems [5, 7].

However, if rebinding of the interior RBs to the membrane of the inclusion rarely happens, we can model the RB-to-EB conversion system under the contact-dependent hypothesis in a much simpler way. As described in the main text, we consider two different species modeling the number of RBs in a single inclusion, RB_1 and RB_2, which model the number of RBs on the inclusion membrane and in the interior of the membrane, respectively. If we only consider the unbinding of RBs on the membrane, we can describe the RB-to-EB conversion mechanism under the contact-dependent hypotheis with three reactions:

$$\begin{aligned}
&RB_1 \xrightarrow{\alpha RB_1} 2RB_1,\\
&RB_1 \xrightarrow{f(RB_1+RB_2+EB)RB_1} RB_2,\\
&RB_2 \xrightarrow{\beta RB_2} EB.
\end{aligned}$$

The first reaction describes the division of the RBs on the boundary, and the second reaction describes the diffusion for the RBs from the membrane to the interior of the inclusion. The last

reaction models the conversion of an RB to an EB. Importantly, in this model, we assumed that conversion occurs only in the interior of the inclusion and the division occurs only on the inclusion membrane.

Another important modeling part, as discussed in the main text, is the diffusion rate f. RBs bound on the membrane may loose the contact to the membrane when the size of the inclusion is big. By assuming spherical shapes of the RBs and the inclusion, this is simply understood as the contact area of the RB to the membrane is small when the curvature of the inclusion is much greater the curvature of the RB (See Figure 4c (left) of the main text). As experimentally measured in [6], furthermore, the size of the inclusion is seemingly proportional to the number of total chlamydiae. Hence we set the diffusion rate function f as a function of $RB_1 + RB_2 + EB$ and set it as a step function to reproduce the bang-bang control as well (See Figure 4c (right) of the main text).

S.E Correlation coefficient between RB and EB

In this section, we show more details about i) the correlation between RB and EB,

$$\rho(RB(t), EB(t)) = \frac{\mathbb{E}\left(RB(t) - \mathbb{E}(RB(t))(EB(t) - \mathbb{E}(EB(t))\right)}{\sqrt{Var\left(RB(t)\right)Var(EB(t))}},$$

is positive for the size-control model and ii) is negative for the communication model and the contact-dependent model at later time points under suitable parameters.

S.E.1 Positive correlation coefficient for the size-control model

For the size-control model, we show that if the RB-to-EB conversion rate β is small enough and the division rate is bigger than the growth reaction rate $\alpha > \gamma$, then the correlation between RB and EB is positive. For the sake of simplicity, we assume that $M = 2$ in (2). That is, we consider three size bins of RBs so that each RB is classified into either RB_0, RB_1, or RB_2.

We first show that there exist positive constants η and C_i, for $i = 1, 2, \dots, 4$ such that for large enough t we have

$$C_1 e^{-\eta t} \le \mathbb{E}(RB(t)) \le C_2 e^{-\eta t}, \quad \text{and} \quad \mathbb{E}(EB(t)) \le C_3 - C_4 e^{-\eta t}, \tag{15}$$

where $RB(t) = \sum_{i=0}^{2} RB_i(t)$. Define a matrix A as

$$A = \begin{pmatrix} -\beta & 2\alpha & 0 \\ 0 & -(\alpha+\gamma) & 2\alpha \\ 0 & \gamma & -\alpha. \end{pmatrix}$$

Then for $x(t) = (\mathbb{E}(RB_0(t)), \mathbb{E}(RB_1(t)), \mathbb{E}(RB_2(t))))^\top$, we have a system of differential equations $\dot{x}(t) = Ax(t)$, which can be derived from the master equation (3). Note that the eigenvalues of A are $-\gamma$, $\frac{-(2\alpha+\gamma)\pm\sqrt{(2\alpha+\gamma)^2-4\alpha(\alpha-\gamma)}}{2}$. If $\alpha > \gamma$, then all eigenvalues are negative. Hence (15) follows from $\dot{x}(t) = Ax(t)$ and $\mathbb{E}(EB(t)) = \beta\int_0^t \mathbb{E}(RB_0(s))ds$.

Using the master equation (3), we can also derive a differential equation for $\mathbb{E}(RB(t)EB(t))$

$$\begin{aligned}\frac{d}{dt}\mathbb{E}(RB(t)EB(t)) =& \alpha\mathbb{E}(RB(t)EB(t)) - (\alpha+\beta)\mathbb{E}(RB_0(t)EB(t)) \\ &+ \beta\mathbb{E}(RB_0(t)RB(t)) - \beta\mathbb{E}(RB_0(t)).\end{aligned}$$

Note that there exists an interval (t_0, t_1) such that for each $t \in (t_0, t_1)$, we have

$$RB(t) \geq 2 \quad \text{and} \quad RB_1(t) + RB_2(t) \geq 1, \tag{16}$$

with high probability. This yields that for $t \in (t_0, t_1)$

1. $\mathbb{E}(RB_0(t)RB(t)) - \mathbb{E}(RB_0(t)) \geq \mathbb{E}(RB_0(t))$, and
2. $\alpha\mathbb{E}(RB(t)EB(t)) - (\alpha+\beta)\mathbb{E}(RB_0(t)EB(t)) \geq 0$ for sufficiently small β,

with high probability Hence we have that

$$\frac{d}{dt}\mathbb{E}(RB(t)EB(t)) \geq \beta\mathbb{E}(RB_0(t)) - \epsilon \quad \text{for } t \in (t_0, t_1), \tag{17}$$

where ϵ shows up due to the small probability that (16) does not hold. Then this implies that $\mathbb{E}(RB(t)EB(t)) \geq C_5 - C_6e^{-\eta t}$ for some positive constants C_5 and C_6 by (15). Finally it follows that the covariance between RB and EB is for $t \in (t_0, t_1)$

$$\mathbb{E}(RB(t)EB(t)) - \mathbb{E}(RB(t))\mathbb{E}(EB(t)) \geq a - be^{-\eta t} - ce^{-2\eta t},$$

for some positive constants a, b and c. Hence we have a positive correlation coefficient between RB and EB at t if we choose parameters so that $t \in (t_0, t_1)$ is large enough.

Remark S.E.1 If either the conversion rate β is small enough or the growth rate γ is not too small, $(t_0, t_1) \subset [12, 40]$. In particular, for the parameters we used to reproduce the mean dynamics as shown in Figure 1d, we have $(t_0, t_1) \subset [12, 40]$. Also (16) holds for most of the time points within $[12, 40]$ under suitable paramters.

S.E.2 Negative correlation coefficient for the communication model

To show the negative correlation between RB and EB numbers at later time points for the communication model, we need to calculate the moments $\mathbb{E}(RB(t)EB(t))$. Due to the step function $f(RB)$, however, it is complicated to compute the moment in a closed form. Hence we roughly verify that the sample points $(RB(t), EB(t))$ are distributed about a straight line with a negative slope on the plane where the x and y axes are RB and EB numbers, respectively. Let $X(t) = (RB(t), EB(t))$ and $\widetilde{X}(t) = (\widehat{RB}(t), \widehat{EB}(t))$ be the two independent Markov chains modeling the communication model with the same parameters.

Let $\tau = \inf\{t > 0 : EB(t) = EB^*\}$ and $\hat{\tau} = \inf\{t > 0 : \widehat{EB}(t) = EB^*\}$ be the time for triggering the promotion of the conversion of the two processes, respectively. For the sake of simplicity, we approximate the $X(t)$ and $\hat{X}(t)$ with the deterministic counterpart of the system, which is

$$\frac{d}{dt}x(t) = (\alpha - f(y(t)))x(t), \quad \frac{d}{dt}y(t) = f(y(t))x(t),$$

where $x(t)$ and $y(t)$ models the concentration (or the mean-field limit) of RB and EB in the communication model, and f is the signal function (9). Hence for $t > \max\{\tau, \hat{\tau}\}$, we have

$$x(t) = x(\tau)e^{(\alpha-\beta')(t-\tau)}, \quad y(t) = EB^* + \frac{\beta'}{\alpha-\beta'}x(\tau)\left(e^{(\alpha-\beta')(t-\tau)} - 1\right)$$
$$\hat{x}(t) = \hat{x}(\hat{\tau})e^{(\alpha-\beta')(t-\hat{\tau})}, \quad \hat{y}(t) = EB^* + \frac{\beta'}{\alpha-\beta'}\hat{x}(\hat{\tau})\left(e^{(\alpha-\beta')(t-\hat{\tau})} - 1\right),$$

where $x(t), y(t), \hat{x}(t)$ and $\hat{y}(t)$ estimate the $RB(t), EB(t), \widehat{RB}(t)$ and $\widehat{EB}(t)$, respectively. We assume that $x(0) = 1$ and $y(0) = 0$ as $RB(0) = 1$ and $EB(0) = 0$. Then for fixed $t > \max\{\tau, \hat{\tau}\}$, the slope of the straight line connecting $X(t)$ and $\hat{X}(t)$ can be approximated with the deterministic values so that the slope is approximately

$$\frac{\hat{y}(t) - y(t)}{\hat{x}(t) - x(t)} = \frac{\alpha-\beta}{\beta}\left(1 + \frac{1}{e^{(\alpha-\beta')t}}\frac{x(\tau) - \hat{x}(\hat{\tau})}{\hat{x}(\hat{\tau})e^{(\beta'-\alpha)\hat{\tau}} - x(\tau)e^{(\beta'-\alpha)\tau}}\right).$$

This quantity is positive only if either $\{\hat{\tau} > \tau\} \cap \{x(\tau) > \hat{x}(\hat{\tau})\}$ or $\{\hat{\tau} < \tau\} \cap \{x(\tau) < \hat{x}(\hat{\tau})\}$. Due to the computational complexity, rather than showing that the probabilities of these events

$$P\left(\{\hat{\tau} > \tau\} \cap \{RB(\tau) > \widehat{RB}(\hat{\tau})\}\right) + P\left(\{\hat{\tau} < \tau\} \cap \{RB(\tau) < \widehat{RB}(\hat{\tau})\}\right),$$

are small, we use Poisson processes with the rate $x(\tau)$ and $x(\hat{\tau})$ to see how likely a positive slope is formed with samples of (RB, EB). Note that RBs are produced by Poissons processes as shown (6). Note also that $x(t)$ and $\hat{x}(t)$ model the average of the numbers of RB and $\widehat{RB}$.

Let Y and $\hat{Y}$ follow Poisson distributions with the rates $x(\tau) = (\alpha-\beta)e^{(\alpha-\beta)\tau}$ and $\hat{x}(\hat{\tau}) = (\alpha-\beta)e^{(\alpha-\beta)\hat{\tau}}$, respectively. Then given $\hat{\tau} > \tau$, $Y - \hat{Y}$ follows the Skellam distribution [8] so that

$$P(Y \geq \hat{Y}) = e^{-x(\tau)-\hat{x}(\hat{\tau})}\sum_{k=0}^{\infty}\left(\frac{x(\tau)}{\hat{x}(\hat{\tau})}\right)^k I_k(2\sqrt{x(\tau)\hat{x}(\hat{\tau})}), \quad \text{where}$$
$$I_k(x) = \sum_{m=0}^{\infty}\frac{1}{m!\Gamma(m+k+1)}\left(\frac{x}{2}\right)^{2m+k} \quad \text{is a modified Bessel function.}$$

As $I_k(2\sqrt{x(\tau)\hat{x}(\hat{\tau})}) \leq c\frac{x(\tau)\hat{x}(\hat{\tau})^{k/2}}{k!}$ for some c independent on $k, x(\tau)$, and $\hat{x}(\hat{\tau})$, we have

$$P(Y \geq \hat{Y}) \leq ce^{-x(\tau)-\hat{x}(\hat{\tau})}\sum_{k=0}^{\infty}\frac{(x(\tau)^{3/2}\hat{x}(\hat{\tau})^{-1/2})^k}{k!} = e^{\frac{x(\tau)}{2} - \frac{3\hat{x}(\hat{\tau})}{2}},$$

which is small if $\hat{x}(\hat{\tau}) = (\alpha-\beta)e^{(\alpha-\beta)\hat{\tau}} > x(\tau) = (\alpha-\beta)e^{(\alpha-\beta)\tau}$(i.e. $\hat{\tau} > \tau$).

S.F Non-monotonic behavior of the EB coefficient for the communication model

The time evolutions of the EB coefficient of variation in the communication model and the contact-dependent model show a non-monotonic behavior around the conversion onset time as shown in

Figure 6a. In this section, by using the communication model, we show which parameter choice causes such a non-monotonic behavior in the EB coefficient of variation. The non-monotonic behavior in the contact-dependent model can be studied similarly.

First, we show the exponential convergence of $CV(\widetilde{EB}(t)) := \frac{(Var(\widetilde{EB}(t)))^{1/2}}{\mathbb{E}(\widetilde{EB}(t))}$ where $\widetilde{X} = (\widetilde{RB}, \widetilde{EB})$ is the associated Markov chain for

$$\widetilde{RB} \xrightarrow{\alpha} 2\widetilde{RB}, \quad \widetilde{RB} \xrightarrow{\beta} \widetilde{EB}, \tag{18}$$

which is a signal-free model. Then the moment equation (13) yields that for each t

$$\mathbb{E}(\widetilde{EB}(t)) = \mathbb{E}(\mathbb{E}_{\widetilde{X}(t_0)}(\widetilde{EB}(t-t_0)) = \bar{b}_1 + \frac{\beta \bar{a}_1}{\alpha - \beta}\left(e^{(\alpha-\beta)(t-t_0)} - 1\right), \tag{19}$$

$$\begin{aligned}\mathbb{E}(\widetilde{EB}^2(t)) &= \mathbb{E}(\mathbb{E}_{\widetilde{X}(t_0)}(\widetilde{EB}^2(t-t_0)) \\ &= \bar{b}_2 + 2\int_{t_0}^{t}\left(\frac{\beta^2\bar{a}_2}{\beta-\alpha} - \frac{(\alpha+\beta)\beta^2\bar{a}_1}{(\beta-\alpha)^2} + \frac{2\alpha\beta^2\bar{a}_1 s}{\beta-\alpha} + \beta\bar{b}_1\bar{a}_1\right)e^{(\alpha-\beta)s}ds \\ &+ 2\int_{t_0}^{t}\left(-\frac{\beta^2\bar{a}_2}{\beta-\alpha} + \frac{(\alpha+\beta)\beta^2\bar{a}_1}{(\beta-\alpha)^2}\right)e^{2(\alpha-\beta)s}ds + \mathbb{E}(\widetilde{EB}(t)),\end{aligned} \tag{20}$$

where t_0 is a fixed time smaller than t, and $\mathbb{E}(\widetilde{RB}(t_0)) = \bar{a}_1, \mathbb{E}(\widetilde{RB}^2(t_0)) = \bar{a}_2, \mathbb{E}(\widetilde{EB}(t_0)) = \bar{b}_1$ and $\mathbb{E}(\widetilde{EB}^2(t_0)) = \bar{b}_2$. Hence both $\mathbb{E}(\widetilde{EB}^2(t))$ and $\mathbb{E}(\widetilde{EB}(t))^2$ are of order $e^{2(\alpha-\beta)t}$ as $t \gg 1$. This implies that

$$\lim_{t\to\infty}\sqrt{\frac{\mathbb{E}(\widetilde{EB}^2(t)) - \mathbb{E}(\widetilde{EB}(t))^2}{\mathbb{E}(\widetilde{EB}(t))^2}} = \lim_{t\to\infty}\sqrt{\frac{\mathbb{E}(\widetilde{EB}^2(t))}{\mathbb{E}(\widetilde{EB}(t))^2} - 1} = c$$

for some constant $c > 0$, and this convergence is exponentially fast. Hence for a small error $\epsilon \ll 1$, we can choose t^* such that $|CV(\widetilde{EB}(t)) - c| < \epsilon$ for each $t > t^*$.

As shown in the main text, our goal is to choose t_1 and t_2 so that (Eq 1) in the main text holds. Now, we show the following proposition.

Proposition S.F.1. *Let $X(t) = (RB(t), EB(t))$ be the stochastic process modeling the communication model (4), with a step function f such that $f(x) = \beta$ for $x < EB^*$ and $f(x) = \beta'$ for $x \geq EB^*$ with $\beta' > \alpha > \beta$. Let $\widetilde{X}(t) = (\widetilde{RB}(t), \widetilde{EB}(t))$ be the stochastic process modeling the reaction network in (18). Suppose that we choose α, β' and EB^* so that for a sufficiently small β, we can select t_2 such that $\mathbb{E}((t_2-\tau)e^{(\alpha-\beta')(t_2-\tau)}\mathbb{1}_{t_2>\tau})$ is small enough and*

$$\mathbb{E}\left(RB(\tau)\left(\frac{\beta'^2}{(\beta'-\alpha)^2}RB(\tau) - \frac{\beta'^2(\beta'+\alpha)}{(\beta'+\alpha)^3} + \frac{4+\beta'}{\beta'-\alpha} - \frac{1+\beta'}{\beta'-\alpha}EB^*\right)\mathbb{1}_{t_2>\tau}\right) > 0 \tag{21}$$

Then there exists a sufficiently small β such that we have

$$\frac{\mathbb{E}(EB^2(t_2))}{\mathbb{E}(EB(t_2))^2} - \frac{\mathbb{E}(\widetilde{EB}^2(t_2))}{\mathbb{E}(\widetilde{EB}(t_2))^2} > 0. \tag{22}$$

Remark S.F.1. (22) implies that

$$CV(EB(t_2)) = \sqrt{\frac{\mathbb{E}(\widetilde{EB}^2(t_2))}{\mathbb{E}(\widetilde{EB}(t_2))^2} + \frac{\mathbb{E}(EB^2(t_2))}{\mathbb{E}(EB(t_2))^2} - \frac{\mathbb{E}(\widetilde{EB}^2(t_2))}{\mathbb{E}(\widetilde{EB}(t_2))^2} - 1}$$
$$> \sqrt{\frac{\mathbb{E}(\widetilde{EB}^2(t_2))}{\mathbb{E}(\widetilde{EB}(t_2))^2} - 1} > CV(\widetilde{EB}(t_2)).$$

Then for an error $\epsilon \ll 1$, if $t_2 > t^*$, then $CV(EB(t_2)) > CV(\widetilde{EB}(t_2)) > c - \epsilon > CV(\widetilde{EB}(t_1)) - 2\epsilon$ due to the exponentially fast convergence of $CV(\widetilde{EB}(t))$ shown above.

Remark S.F.2. (21) is not a restrictive condition, as $RB(t)$ grows much faster than $EB(t)$ for $t < \tau$ if we choose $\alpha > \beta$ with a small enough β. Hence we expect $RB(\tau)$ is much greater than $EB(\tau)$.

Proof of Proposition S6.1. Let $\tau = \inf\{t > 0 : EB(t) = EB^*\}$. We let $RB^* = \mathbb{E}(RB(\tau))$ and $R^{**} = \mathbb{E}(RB^2(\tau))$. Note that due to $EB(t) = \widetilde{EB}(t)$ for $t < \tau$, we have $a = \mathbb{E}(\widetilde{EB}(t_2)\mathbb{1}_{t_2<\tau})$ and $b = \mathbb{E}(\widetilde{EB}(t_2)^2\mathbb{1}_{t_2<\tau})$.

To show (22), if suffices to show that

$$\mathbb{E}(EB^2(t_2))\mathbb{E}(\widetilde{EB}(t_2))^2 - \mathbb{E}(\widetilde{EB}^2(t_2))\mathbb{E}(EB(t_2))^2 > 0. \tag{23}$$

We first consider a decomposition of $\mathbb{E}(EB(t)) = \mathbb{E}(EB(t)\mathbb{1}_{t\geq\tau}) + \mathbb{E}(EB(t)\mathbb{1}_{t<\tau})$. Using conditional expectations, we have

$$\begin{aligned}\mathbb{E}(EB(t)\mathbb{1}_{t>\tau}) &= \mathbb{E}(\mathbb{E}(EB(t)|\mathcal{F}_\tau)\mathbb{1}_{t>\tau}) = \mathbb{E}(\mathbb{E}_{X(\tau)}(EB(t-\tau))\mathbb{1}_{t>\tau})\\ \mathbb{E}(EB^2(t)\mathbb{1}_{t>\tau}) &= \mathbb{E}(\mathbb{E}(EB^2(t)|\mathcal{F}_\tau)\mathbb{1}_{t>\tau}) = \mathbb{E}(\mathbb{E}_{X(\tau)}(EB^2(t-\tau))\mathbb{1}_{t>\tau}),\end{aligned} \tag{24}$$

where $\mathcal{F}_\tau$ is the sigma algebra with respect to τ. Hence we first explore $\mathbb{E}_{X(\tau)}(EB(t-\tau))$ and $\mathbb{E}_{X(\tau)}(EB^2(t-\tau))$ for $t > \tau$ regarding τ as a fixed time. Then with the same derivation for (25) and (26), we have

$$\mathbb{E}_{X(\tau)}(EB(t-\tau)) = EB^* - \frac{\beta' RB^*}{\beta' - \alpha}\left(e^{(\alpha-\beta')(t-\tau)} - 1\right), \tag{25}$$

$$\begin{aligned}\mathbb{E}_{X(\tau)}(EB^2(t-\tau)) = {} & (EB^*)^2 - EB^* + \mathbb{E}_{X(\tau)}(EB(t-\tau))\\ & + 2\beta'\Bigg(\int_0^{t-\tau}\left(\frac{\beta'(\beta'+\alpha)}{(\beta'+\alpha)^2}RB^* - \frac{\beta'}{(\beta'-\alpha)}RB^{**}\right)e^{-2(\beta'-\alpha)s}ds\\ & - \int_0^{t-\tau}\left(\frac{\beta'(\beta'+\alpha)}{(\beta'-\alpha)^2}RB^* - \frac{1}{\beta'-\alpha}RB^{**}\right)e^{-(\beta'-\alpha)s}ds\\ & + \int_0^{t-\tau}\left(\frac{2\beta'}{\beta'-\alpha}sRB^* + RB^*EB^*\right)e^{-(\beta'-\alpha)s}ds\Bigg).\end{aligned} \tag{26}$$

By computing those intergrations we obtain that

$$\begin{aligned}\mathbb{E}_{X(\tau)}(EB^2(t-\tau)) = {} & (EB^*)^2 - EB^* + \mathbb{E}_{X(\tau)}(EB(t-\tau))\\ & + \frac{\beta'^2}{(\beta'-\alpha)^2}RB^{**} - \frac{\beta'^2(\beta'+\alpha)}{(\beta'-\alpha)^3}RB^* - \frac{4\beta'^2}{(\beta'-\alpha)^3}RB^* - \frac{RB^*EB^*}{\beta'-\alpha}\\ & - \frac{4\beta'^2}{(\beta'-\alpha)^2}(t-\tau)e^{-(\beta'-\alpha)(t-\tau)}RB^*.\end{aligned}$$

Here, as we assumed in the theorem statement, we can neglect the term $\frac{4\beta'^2}{(\beta'-\alpha)^2}(t_2-\tau)e^{-(\beta'-\alpha)(t_2-\tau)}RB^*$. Similarly, from (19) and (20) we can obtain the same equations for $(\widetilde{RB}, \widetilde{EB})$ as (25) and (26) but β' replaced with β. Then the term $\frac{\beta' RB^*}{\beta'-\alpha}$ in (25) and the integration terms in (26) with β' replaced with β are negligible if β is small enough for a fixed t_2. Hence we have estimations of

$$\mathbb{E}_{\widetilde{X}(\tau)}(\widetilde{EB}(t_2-\tau)) \approx EB^*, \tag{27}$$

$$\mathbb{E}_{\widetilde{X}(\tau)}(\widetilde{EB}^2(t_2-\tau)) \approx (EB^*)^2 - EB^* + \mathbb{E}_{X(\tau)}(EB(t_2-\tau)). \tag{28}$$

Then we finally have

$$\begin{aligned}
\mathbb{E}(EB(t_2)\mathbb{1}_{t_2>\tau}) &= EB^*P(t_2>\tau) - \frac{\beta'}{\beta'-\alpha}\mathbb{E}\left(\left(e^{(\alpha-\beta')(t_2-\tau)}-1\right)\mathbb{1}_{t_2>\tau}\right), \\
\mathbb{E}(EB^2(t_2)\mathbb{1}_{t_2>\tau}) &= \mathbb{E}(\mathbb{E}_{X(\tau)}(EB^2(t_2-\tau)\mathbb{1}_{t_2>\tau}) \\
&\approx ((EB^*)^2 - EB^*)P(t_2>\tau) + \mathbb{E}(\mathbb{E}_{X(\tau)}(EB(t-\tau))) \\
&\quad + \frac{\beta'^2}{(\beta'-\alpha)^2}\mathbb{E}(RB^2(\tau)\mathbb{1}_{t_2>\tau}) - \frac{\beta'^2(\beta'+\alpha)}{(\beta'-\alpha)^3}\mathbb{E}(RB(\tau)\mathbb{1}_{t_2>\tau}) \\
&\quad - \frac{4\beta'^2}{(\beta'-\alpha)^3}\mathbb{E}(RB(\tau)\mathbb{1}_{t_2>\tau}) - \frac{EB^*}{\beta'-\alpha}E(RB(\tau)\mathbb{1}_{t_2>\tau}) \\
\mathbb{E}((\widetilde{EB}(t_2))\mathbb{1}_{t_2>\tau}) &\approx EB^*P(t_2>\tau), \\
\mathbb{E}(\widetilde{EB}^2(t_2)\mathbb{1}_{t_2>\tau}) &\approx ((EB^*)^2 - EB^*)P(t_2>\tau) + \mathbb{E}(\mathbb{E}_{X(\tau)}(EB(t_2-\tau))),
\end{aligned}$$

as $\beta \to 0$. Then (22) follows since (23) is equivalent to (21) as $\beta \to 0$. □

Remark S.F.3. It is also critical to show that we can select t_1 satisfying $t_1 < t_2$. Note that by (20) the convergence rate of $\sqrt{\frac{\mathbb{E}(\widetilde{EB}^2(t)) - \mathbb{E}(\widetilde{EB}(t))^2}{\mathbb{E}(\widetilde{EB}(t))^2}}$ is determined by the convergence of $e^{(\alpha-\beta)t}$ as $t \to \infty$. Also, the average value of τ can be controlled by α and β. Using these facts, we can show that it is possible to select $t_1 < t_2$ if α is sufficiently large and β is sufficiently small. In such a case, even for small enough t_1, the term $e^{(\alpha-\beta)t}$ is sufficiently large so that the coefficient of variation of $\widetilde{EB}$ is about the convergent value at t_1.

A large value of α leads more RBs produced and hence more EBs may be expected to be produced, which in turn makes τ small. However, for sufficiently small β, conversion to EB can be delayed enough so that we can still keep τ not too small. As t_2 is supposed to be around the conversion onset time τ, this finally allows us to select t_1 smaller than t_2.

S.G Choice of system parameters

In this section, we demonstrate that the statistical features used to test the three models are robust to parameter selection. Additionally, we provide further details on model-fitting to experimental data sets using the simulated annealing algorithm.

S.G.1 Robustness of the statistical features to parameters that induce the bang-bang behavior

For the communication and contact-dependent models, we illustrated in the main text the causes of the negative correlation at later time points and the non-monotonic behavior of the EB coefficient of variation. For example, in the communication model, RBs quickly convert to EBs after the EB number reaches the threshold, while RBs divide and rarely convert before the threshold is reached. This runaway conversion induces the bang-bang behavior of RBs in the mean number of RBs. Regarding variational behavior, the quick conversion causes data points based on RB and EB counts to align along a negatively sloped line, indicating a negative RB-EB correlation. Furthermore, this runaway conversion means that inclusions reaching the threshold slightly earlier than others will contain significantly more EBs than average, leading to an increase in the EB coefficient of variation. Thus, we anticipate that these statistical features will remain robust for any parameter choice, provided that the bang-bang behavior occurs with rapid RB-to-EB conversion triggered by an extrinsically controlled threshold.

To validate this, in Figure B, we display the time evolution of the RB-EB correlation and the EB coefficient of variation in the communication model under different parameter choices. Notably, under these parameters, the RB number exhibits bang-bang behavior (Figure B, top), though this does not provide the best fit with experimental measurements. As shown in Figure B, none of the parameter sets produce either a positive RB-EB correlation at all time points or a monotonic EB coefficient of variation (Figure B, middle and bottom). Similarly, for the contact-dependent model under the parameter choices used in Figure B, we showed that the bang-bang behavior is reproduced (Figure 3D and Figure B, top), but neither the RB-EB correlation nor the EB coefficient aligns with experimental measurements (Figure B, middle and bottom).

Next, we demonstrate that over a broad range of parameters, neither the communication model nor the contact-dependent model can exhibit a positive RB-EB correlation at all time points nor a monotonically decreasing EB coefficient of variation while maintaining bang-bang control in the RB number. We used 132401 parameter sets in which the division rate is lower than the conversion rate after reaching the threshold. This restriction is essential for the system to achieve rapid RB-to-EB conversion. For each parameter set, we ran the stochastic simulation algorithm and evaluated three criteria:

- C1 the local maximum of $RB(t)$ occurs at $t \in [20, 35)$ h.p.i., $RB(t) < 5000$ for each t, and $EB(40) > RB(40)$,
- C2 the RB-EB correlation remains non-negative (i.e. $\min_{t \in [12,40]} \rho(RB(t), EB(t)) > 0$),
- C3 the EB coefficient of variation does not significantly increase (i.e. $CV(EB(t+1)) - CV(EB(t)) < 0.1$ for each $t \in [12, 40]$).

Specifically, C1 ensures that the RB number exhibits bang-bang behavior observed in the experimental data sets.

The division rate α ranges from 0.1 to 1 across the 132401 parameter sets for each of the two models. If the division rate exceeds 1, the RBs divide too frequently by $t = 30$ hpi (for example, if $\alpha = 1$, RBs divide once per unit hpi, leading to an approximate RB count of $2^{30-12} = 2^{18}$ by $t = 30$ hpi). If $\alpha < 0.1$, each RB divides once every 10 hpi on average, which is too rare to reproduce the

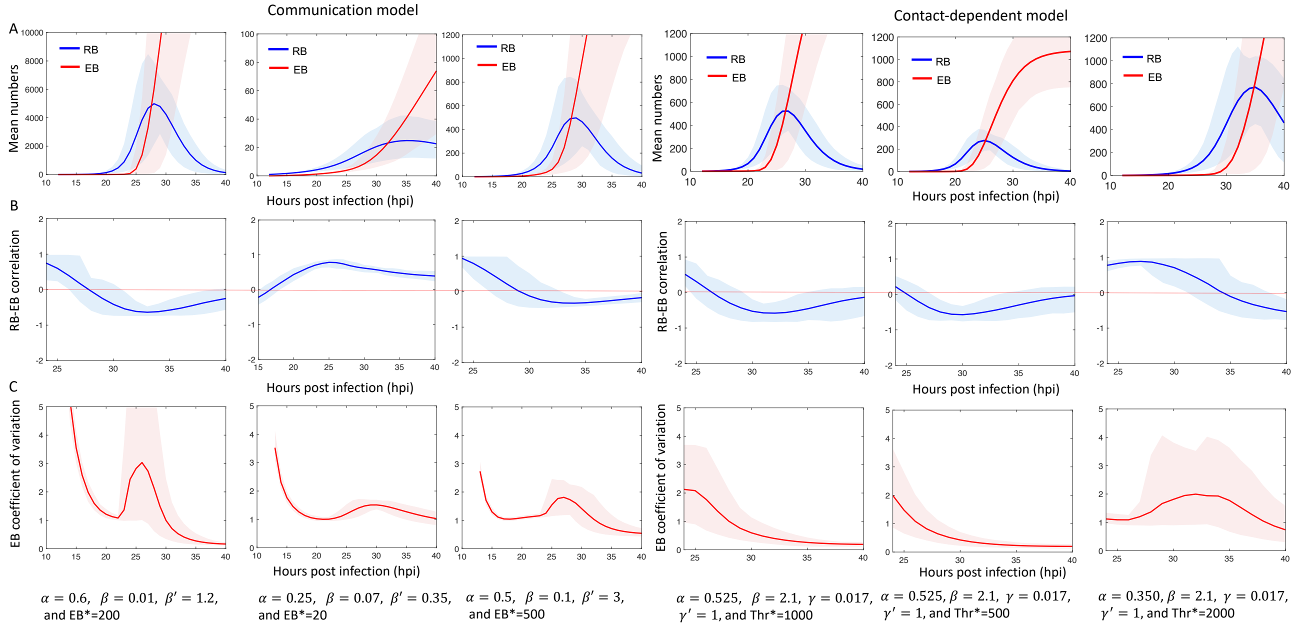

Figure B: The bang-bang behavior (A) of the communication and the contact-dependent models under various choices of parameters, and the corresponding RB-EB correlations (B) and the EB coefficients of variation (C). Each column corresponds to the parameter set specified at the bottom. Notably the RB-EB correlation is positive at later time points, while it is negative at an earlier time point in the second plot of B. This is because β' is not significantly larger than α causing a non-substantial decrease of the RB number at the later time points. Furthermore since $\alpha - \beta$ is not big enough, the data points (RB, EB) may not be clearly aligned on a negatively sloped straight line as analyzed in Section S.E.2.

experimental result. Therefore, division rates outside the range $[0.1, 1]$ are excluded to better align with experimental measurements. Similarly, the conversion rate β' is set between 0.1 and 3 for both models. For the contact-dependent model, the diffusion rate γ' after reaching the threshold is also set between 0.1 and 3. Finally, the threshold ranges from 20 to 500 for the communication model and from 500 to 1000 for the contact-dependent model, as thresholds beyond these values are unrealistic relative to experimental data. From these ranges, we evenly select 132401 parameter sets where the conversion rate β' is greater than the division rate α for the communication model and the contact-dependent model.

Among these parameter sets, none satisfy both C1 and C2 for either the communication model or the contact-dependent model. This indicates that neither model can reproduce the positive RB-EB correlation within $t \in [12, 40)$ over a broad range of biologically reasonable parameter sets. Similarly, none of the parameter sets allow either model to meet both C1 and C3. This suggests that neither model can exhibit a monotonic decrease in the time evolution of the EB coefficient under reasonable parameter choices, in contrast to experimental measurements.

We repeated a similar test for the size control model. For the division and conversion rates, we evenly selected them from the interval $[0.1, 1]$. If the rates fall outside this range, RBs disappear too quickly. The range of growth is from 0.001 to 0.1; otherwise, too many RBs would replicate. We also evenly selected the minimum number of divisions for conversion (M) between 5 and 15.

Combining these parameter choices, we tested $88,000$ different parameter sets with the size control model and found that it satisfies C1, C2, and C3 simultaneously for $8,854$ parameter sets out of the $80,000$ tested. Consequently, this parameter search for the three models supports our main claim.

S.G.2 Pseudo codes for the simulated annealing algorithm

In this section, we show pseudo codes for the simulated annealing algorithm that we used to search the parameters. See Method in the main text for the basic setting for the simulated annealing algorithm. Recall that we used a loss function defined as

$$L(RB, EB) = \sum_{i=1}^{8} \left(|RB(t_i) - \hat{RB}(t_i)|^2 + 0.1|EB(t_i) - \hat{EB}(t_i)|^2 \right),$$

where (RB, EB) and $(\hat{RB}, \hat{EB})$ are the time trajectories of RB and EB obtained with the model simulation and the experimental measurements. Let $(RB_{\text{new}}, EB_{\text{new}})$ be the time trajectories obtained with the new parameters. Then $\Delta L = L(RB_{\text{new}}, EB_{\text{new}}) - L(RB, EB)$ can be used to define the acceptance probability $p = exp(-\Delta L/T)$. We set the temperature parameter $T = 1$. Then we accept the new parameters with the acceptance probability p. We repeated this 1000 times. This is the pseudo-code.

$T \leftarrow 1$ and $k \leftarrow 1$
while $k \leq 1000$ **do**
  $x_{\text{new}} \leftarrow x + x \cdot U_x$, where $U_x \sim$ Uniform$(-0.2, 0.2)$
  $(RB_{\text{new}}, EB_{\text{new}}) \leftarrow$ simulate(x_{new})
  $\Delta L \leftarrow L(RB_{\text{new}}, EB_{\text{new}}) - L(RB, EB)$
  $p \leftarrow \exp(-\Delta L/T)$
  if rand$(0, 1) < p$ **then**
    $x \leftarrow x_{\text{new}}$
    $(RB, EB) \leftarrow (RB_{\text{new}}, EB_{\text{new}})$
  end if
  $k \leftarrow k + 1$
end while

S.H Statistical features of the communication model with EB-derived positive feedback modeled by a smooth signal, $f(EB)$.

For the communication model with positive feedback given by $EB(t)$ for the RB-to-EB conversion, we can set the signal function $f(EB)$ to be a smooth function rather than the step function (Figure 3c). As shown Figure C, the growth curve, the RB-EB correlation, and the coefficient of variation of $EB(t)$ have similar qualitative behaviors as the case when $f(EB)$ is a step function. This highlights the generality of our modeling.

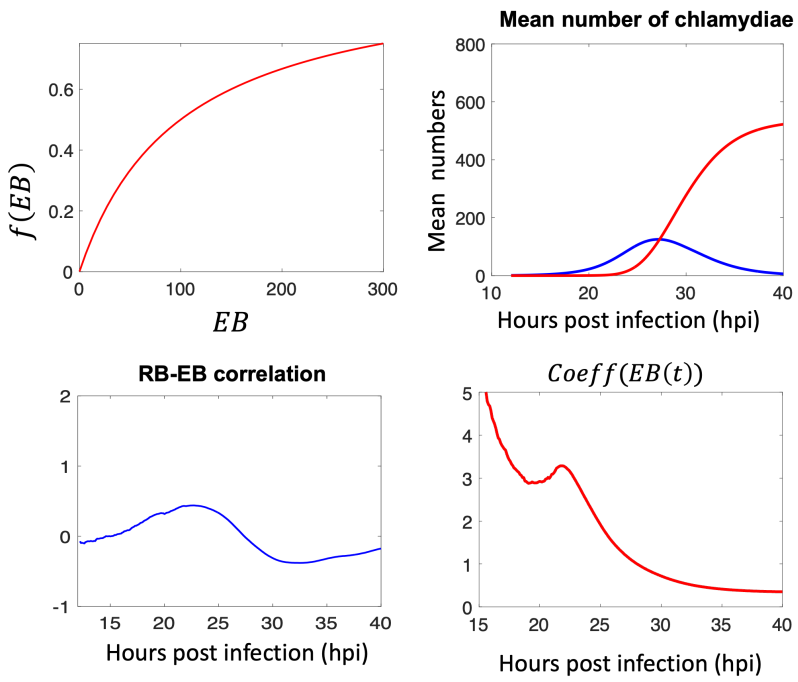

Figure C: The time evolutions of the statistical features of the communication model with a smooth extrinsic signal.

References

[1] David F. Anderson and Thomas G Kurtz. Continuous time Markov chain models for chemical reaction networks. In H Koeppl Et al., editor, *Design and Analysis of Biomolecular Circuits: Engineering Approaches to Systems and Synthetic Biology*, pages 3–42. Springer, 2011.

[2] David F. Anderson and Thomas G. Kurtz. *Stochastic analysis of biochemical systems*, volume 1.2 of *Stochastics in Biological Systems*. Springer International Publishing, Switzerland, 1 edition, 2015.

[3] EB Dynkin. Markov processes, vol. ii. translated with the authorization and assistance of the author by j. fabius, v. greenberg, a. maitra, g. majone. *Die Grundlehren der Mathematischen Wissenschaften*, 122, 1965.

[4] Thomas G Kurtz. Representations of Markov Processes as Multiparameter Time Changes. *The Annals of Probability.*, 8(4):682–715, 1980.

[5] SD Lawley and JB Madrid. First passage time distribution of multiple impatient particles with reversible binding. *The Journal of chemical physics*, 150(21), 2019.

[6] Jennifer K Lee, Germán A Enciso, Daniela Boassa, Christopher N Chander, Tracy H Lou, Sean S Pairawan, Melody C Guo, Frederic YM Wan, Mark H Ellisman, Christine Sütterlin, et al. Replication-dependent size reduction precedes differentiation in chlamydia trachomatis. *Nature communications*, 9(1):45, 2018.

[7] Albert Libchaber. From biology to physics and back: The problem of brownian movement. *Annual Review of Condensed Matter Physics*, 10:275–293, 2019.

[8] John G Skellam. The frequency distribution of the difference between two poisson variates belonging to different populations. *Journal of the Royal Statistical Society Series A: Statistics in Society*, 109(3):296–296, 1946.
