# Supplementary material for: Statistical analysis supports the size control mechanism of Chlamydia development
Source: PLoS Comput Biol. 2025 Jul 14;21(7):e1013227. doi: 10.1371/journal.pcbi.1013227 (PMC12279117; doi:10.1371/journal.pcbi.1013227)
Supplement: S1 Table — (PDF) [file pcbi.1013227.s002.pdf]

Inclusion	hpi	RB+DB	EB+IB	Inclusion	hpi	RB+DB	EB+IB	Inclusion	hpi	RB+DB	EB+IB
1	12	1	0	51	16	14	0	104	24	79	0
2	12	1	0	52	16	3	0	105	24	155	18
3	12	1	0	53	16	9	0	106	24	63	0
4	12	1	0	54	16	2	0	107	24	77	0
5	12	2	0	55	16	5	0	108	24	187	16
6	12	1	0	56	16	6	0	109	24	79	3
7	12	1	0	57	16	5	0	110	24	280	0
8	12	1	0	58	16	6	0	111	24	44	0
9	12	2	0	59	16	12	0	112	24	33	0
10	12	1	0	60	16	2	0	113	24	52	0
11	12	1	0	61	16	18	0	114	28	531	140
12	12	1	0	62	16	8	0	115	28	152	0
13	12	2	0	63	16	4	0	116	28	502	49
14	12	1	0	64	16	13	0	117	28	142	0
15	12	1	0	65	16	8	0	118	28	324	138
16	12	2	0	66	16	8	0	119	28	234	212
17	12	2	0	67	16	5	0	120	28	800	472
18	12	2	0	68	16	2	0	121	28	366	184
19	12	1	0	69	16	8	0	122	28	303	129
20	12	1	0	70	16	21	0	123	28	190	63
21	12	2	0	71	16	14	0	124	28	55	0
22	12	1	0	72	16	8	0	125	28	1090	925
23	12	1	0	73	16	8	0	126	28	320	186
24	12	1	0	74	16	7	0	127	32	1049	1609
25	12	1	0	75	16	8	0	128	32	440	651
26	12	1	0	76	16	4	0	129	32	700	721
27	12	1	0	77	16	2	0	130	32	148	89
28	12	1	0	78	16	8	0	131	32	801	1413
29	12	1	0	79	16	8	0	132	32	502	773
30	12	1	0	80	16	4	0	133	32	284	295
31	12	1	0	81	16	7	0	134	32	376	86
32	12	1	0	82	20	13	0	135	32	533	644
33	12	2	0	83	20	2	0	136	32	238	282
34	12	1	0	84	20	26	0	137	36	223	293
35	12	2	0	85	20	45	0	138	36	272	594
36	12	1	0	86	20	50	0	139	36	397	1077
37	12	1	0	87	20	16	0	140	36	171	367
38	12	1	0	88	20	54	0	141	36	196	471
39	12	2	0	89	20	26	0	142	36	158	379
40	12	1	0	90	20	29	0	143	36	310	1105
41	12	1	0	91	20	61	0	144	36	602	1842
42	12	1	0	92	20	40	0	145	36	105	228
43	12	1	0	93	20	61	0	146	40	184	1773
44	12	2	0	94	20	26	0	147	40	190	768
45	12	1	0	95	20	24	0	148	40	51	81
46	12	1	0	96	20	18	0	149	40	92	189
47	12	1	0	97	20	44	0	150	40	319	1065
48	12	2	0	98	20	58	0	151	40	267	2051
49	12	1	0	99	20	84	0	152	40	115	133
50	12	2	0	100	20	13	0	153	40	203	642
				101	20	29	0	154	40	121	334
				102	20	8	0	155	40	163	472
				103	20	15	0				
